# H5N1 clade 2.3.4.4b avian influenza viruses replicate in differentiated bovine airway epithelial cells cultured at air-liquid interface

**DOI:** 10.1099/jgv.0.002007

**Published:** 2024-06-26

**Authors:** Luca Bordes, Nora M. Gerhards, Stan Peters, Sophie van Oort, Marit Roose, Romy Dresken, Sandra Venema, Manouk Vrieling, Marc Engelsma, Wim H.M. van der Poel, Rik L. de Swart

**Affiliations:** 1 Wageningen Bioveterinary Research, Lelystad, Netherlands

**Keywords:** air-liquid interface, airway epithelial cells, cattle, cow, H5N1, highly pathogenic avian influenza, ruminants

## Abstract

Highly pathogenic avian influenza (HPAI) H5N1 viruses are responsible for disease outbreaks in wild birds and poultry, resulting in devastating losses to the poultry sector. Since 2020, an increasing number of outbreaks of HPAI H5N1 was seen in wild birds. Infections in mammals have become more common, in most cases in carnivores after direct contact with infected birds. Although ruminants were previously not considered a host species for HPAI viruses, in March 2024 multiple outbreaks of HPAI H5N1 were detected in goats and cattle in the United States. Here, we have used primary bronchus-derived well-differentiated bovine airway epithelial cells (WD-AECs) cultured at air-liquid interface to assess the susceptibility and permissiveness of bovine epithelial cells to infection with European H5N1 virus isolates. We inoculated bovine WD-AECs with three low-passage HPAI clade 2.3.4.4b H5N1 virus isolates and detected rapid increases in viral genome loads and infectious virus during the first 24 h post-inoculation, without substantial cytopathogenic effects. Three days post-inoculation infected cells were still detectable by immunofluorescent staining. These data indicate that multiple lineages of HPAI H5N1 may have the propensity to infect the respiratory tract of cattle and support extension of avian influenza surveillance efforts to ruminants. Furthermore, this study underscores the benefit of WD-AEC cultures for pandemic preparedness by providing a rapid and animal-free assessment of the host range of an emerging pathogen.

## Introduction

The evolutionary trajectory of the A/Goose/Guangdong/1/1996 (Gs/Gd) lineage of highly pathogenic avian influenza (HPAI), characterized by reassortments and mutations, led to its diversification into numerous clades [1]. Since 2003 HPAI of the Gs/Gd lineage is enzootic in Asia, causing repeated outbreaks in poultry [2]. A clade 2.2 HPAI H5N1 virus from the Gs/Gd lineage was introduced into Europe in 2005, causing significant mortality amongst wild birds and required culling of numerous infected poultry farms [2,3]. The H5N1 subtype re-emerged in Europe late 2020 and continued to diversify in multiple genotypes, which subsequently caused the largest HPAI epizootic recorded to date [4]. In addition to high mortality in waterfowl and numerous outbreaks in poultry, an increasing number of HPAI H5N1 infections was observed in mammals [5]. The virus continued to spread to North America via the pacific flyway and was detected in the United States of America (USA) by January 2022 in wild birds and February 2022 in poultry [6,7]. Similar to the European epizootic in 2020–2021, the HPAI H5N1 virus caused numerous outbreaks in poultry, mass mortality in wild birds and occasional introductions in mammalian species in the USA [8].

In March 2024, a first HPAI H5N1 infection was observed in ruminants, on a recently depopulated poultry farm in Minnesota, USA [9]. Ten goat kids died from an unknown cause. Five of these animals tested positive for HPAI H5N1 in multiple tissue samples, including the brain. The goats and the recently depopulated poultry shared a sole water source and pasture. Two days after the report in goats, cattle in two dairy farms in Kansas and one in Texas were tested positive for HPAI H5N1 [10]. To date (early May, 2024), 36 dairy farms have tested positive for HPAI H5N1, and the virus is believed to be introduced by a single wild bird-to-cattle transmission event [11]. So far, further transmission between herds is associated with asymptomatic cattle movement. On one location a farmer developed conjunctivitis and was laboratory diagnosed to be infected with HPAI H5N1, which carried the mammalian adaptation PB2-E627K [12]. According to the Centres of Diseases Control and Prevention (CDC), transmission of HPAI H5N1 to humans is possible through direct contact with infected animals, but human-to-human transmission remains unlikely with the current genetic composition of HPAI H5N1 [12]. Analysis of the virus sequences detected in cattle showed the virus is of avian origin (clade 2.3.4.4b, genotype B3.13) and is closely related to wild bird isolates. Several amino acid changes associated with mammalian adaptation were identified in the cattle sequences including the PB2-M631L amino acid change which was present in all cattle isolated viruses [11,13, 14]. The B3.13 genotype contains PA, HA, NA and M gene segments from Eurasian wild bird lineages and PB2, PB1, NP and NS gene segments from American wild bird lineages.

Clinical signs of H5N1 infection in cattle included decreased milk production, reduced feed intake with coinciding drop in rumen motility, lethargy, abnormally loose faeces and fever [10]. Severely affected cows (in particular older animals) experienced colostrum-like milk or acute drop in milk production. Unlike HPAI H5N1 infections in poultry, mortality in cows seemed limited and infected animals recovered after about 7 to 10 days. During the infectious period high viral loads have been detected in milk, while virus shedding from the respiratory tract was limited. This may pose a risk for dairy farmers and unpasteurized milk consumption should be avoided.

Influenza A viruses are not considered to circulate widely in ruminants [15]. However, it is evident that the current North American HPAI H5N1 B3.13 virus is able to surpass the bird-to-ruminant species barrier. This raises the question whether the same is true for other HPAI H5N1 viruses. For a preliminary screening of the susceptibility and permissiveness of bovine airway epithelial cells (AECs) to infection with HPAI, we have performed experimental inoculation of well-differentiated bovine AECs (WD-AECs) grown at air-liquid interface (ALI). These cultures form a pseudostratified ciliated columnar epithelium containing a basal layer and mucus-producing cells and can be used as a bridge between traditional *in vitro* models in immortalized cells and *in vivo* studies in animals [16]. We inoculated bovine WD-AECs with three early passage HPAI clade 2.3.4.4b H5N1 virus isolates. The viruses were isolated in the Netherlands, are closely related to contemporary European HPAI H5N1 viruses and were isolated from mixed host species (poultry, red fox with PB2-627E, red fox with PB2-627K) [17]. All three viruses produced infectious virus in bovine WD-AECs without causing significant damage to the epithelial cell layer.

## Methods

### H5N1 virus isolates

HPAI strain H5N1-2020 (A/chicken/netherlands/20019879-001005/2020, EPI_ISL_17791407) was isolated from a swab sample collected from poultry, while H5N1-2021 PB2-627E (A/red fox/Netherlands/21040099-006_PB2E/2021, EPI_ISL_19057416) and H5N1-2021 PB2-627K (A/red fox/Netherlands/21040099-006_PB2K/2021, EPI_ISL_19057417) were isolated from a brain homogenate of a fox, and subsequently separated by serial dilution as described previously [17]. In-depth analysis of the sequencing data did not reveal other known or previously described host shift adaptations in the fox viruses apart from the PB2-E627K amino acid change [17]. Virus stocks were obtained after two passages in 9- to 11-day-old embryonated Specific Pathogen Free (SPF) chicken eggs (further referred to as E2 passage). Both the seed material and E2 passage were sequenced as described previously [18]. After RNA isolation using the High Pure Viral RNA kit (Roche), viral RNA was amplified using universal eight segment primers. The PCR products were sequenced with an average coverage >1000 per nucleotide position using Illumina DNA Prep and Illumina MiSeq 150PE sequencing. Reads were subsequently mapped to a set of reference genomes using the CLC Genomics Workbench extension ViralProfiler Workflow (Qiagen) before generating consensus sequences which were uploaded to GISAID. No differences were observed between the seed material and E2 passages. Median 50 % egg infective dose (EID_50_) and median 50 % tissue culture infective dose (TCID_50_) of the virus isolates were determined by end-point titration on 9- to 11-day-old SPF embryonated chicken eggs as described previously [18] and on Madin-Darby canine kidney (MDCK) cells, as described below. The virus isolates were titrated in triplicate on different days and EID_50_ titres were calculated using the Spaerman-Kärber algorithm [19,20].

### Phylogenetic analysis

Phylogenetic analysis of H5N1 genome sequences was performed as described previously [17]. Briefly, sequences were aligned using MAFFT v7.475 [21], phylogeny was reconstructed using maximum likelihood analysis with IQ-TREE software v2.0.3 and 1000 bootstrap replicates [22] and subsequently visualized using the R package ggtree [23]. In addition to the strains used in this study, the WHO subclade 2.3.4.4 a, 2.3.4.4b, 2.3.4.4 c and 2.3.4.4d representative strains and a random selected set of H5N1 genome sequences from goat, cattle and other mammals were used for the phylogenetic analysis. The GISAID sequences used in the phylogenetic analysis are listed in Table S1 (available in the online version of this article) [24].

### Isolation of bovine primary bronchial epithelial cells

Bovine lungs were obtained from a local slaughterhouse in the Netherlands on the day of slaughter, and transported on melting ice. The primary bronchi were dissected from the bifurcation to about 10–15 cm distal and cut into fragments of about 3 cm^2^. After two washes in PBS (Thermo Fisher Scientific) containing 1 x antibiotic-antimycotic (Thermo Fisher Scientific), the tissue was digested overnight at 4 °C in Dulbecco’s Modified Eagle Medium/Nutrient Mixture F-12/GlutaMAX (Gibco) containing 10 µg ml^−1^ DNAse (Sigma-Aldrich), 1 mg ml^−1^ protease XIV (Sigma-Aldrich), 1 x antibiotic-antimycotic and 0.1 mg ml^−1^ primocin (InVivogen) on a gentle platform rocker. The next day, loosely attached bronchial epithelial cells were harvested by scraping with a scalpel blade, collected in PBS and passed through a 70 µm cell strainer before centrifugation at 300 ***g*** for 5 min at 4 °C, followed by lysis of erythrocytes using Red Blood Cell Lysis buffer (Roche). After resuspension in PBS and another centrifugation step, the cell pellet was resuspended in DMEM/F12 and aliquots were frozen in DMEM/F12 with 40 % FCS and 10 % DMSO (Honeywell).

### Culture of bovine bronchial epithelial cells

Cryovials containing primary bovine bronchial epithelial cells were quickly defrosted and cells were seeded on T25 tissue culture flasks coated with collagen I (Corning) in Airway Epithelial Cell Basal Medium (AECBM) prepared according to manufacturer’s instructions (Promocell), supplemented with 1 x antibiotic-antimycotic and 0.1 mg ml^−1^ primocin. Cells were cultured at 37 °C and 5 % CO_2_. After expansion to 60–80 % confluency, cells were dissociated using Trypsin/EDTA solution, HEPES buffered saline solution and Trypsin neutralizing solution (all Lonza Bioscience). Then 0.4 µm pore size Nunc Cell Culture inserts in Nunc carrier plates (both Thermo Fisher Scientific), pore density <0.85×10^8^ pores cm^−2^, 0.47 cm^2^ culture area, were coated with collagen IV and fibronectin (both Sigma-Aldrich) before seeding of dissociated primary cells at a density of 1×10^5^–10.5×10^5^ cells per transwell filter in AECBM. Cells were cultured as liquid-liquid interface cultures at 37 °C and 5 % CO_2_. After 5–10 days a tight monolayer had formed, which allowed the removal of cell culture medium from the apical compartment, generating air-liquid interface cultures. The next day, the basolateral medium was exchanged by Airway Epithelial Cell Differentiation medium (AECDM), consisting of AECBM (Promocell) and Dulbecco’s Modified Eagle Medium/High Glucose (Sigma-Aldrich) mixed at a 1 : 1 ratio, and supplemented with 0.004 ng ml^−1^ bovine pituitary extract (BPE), 5 µg ml^−1^ insulin, 0.5 µg ml^−1^ hydrocortisone, 10 µg ml^−1^ transferrin, 6.7 ng ml^−1^ triiodothyronine, 0.5 µg ml^−1^ epinephrine, 10 ng ml^−1^ human epidermal growth factor (hEGF) (all Promocell), 100 nM retinoic acid (Sigma-Aldrich) and 1 x antibiotic-antimycotic. Subsequently, cells were differentiated at air-liquid interface over the course of approximately 4 weeks at 37 °C and 5 % CO_2_. AECBM Medium was exchanged every other day while culturing transwell filters in lowest position in carrier plates. During differentiation, AECDM was exchanged every 5 days, and filters were kept in the medium position of the carrier plate. Once a week, an apical wash was performed using pre-warmed PBS (containing magnesium and calcium) to remove excess mucus.

### H5N1 HPAI infection of differentiated bovine air-liquid interface (ALI) cultures

Eight primary bronchus-derived WD-AEC filters from a single donor animal were used to assess the replication capacity of three low-passage virus isolates, with each virus isolate tested in technical duplicates and two mock-infected control WD-AECs. On the day of virus infection, pre-warmed PBS was added to the apical compartment of the WD-AECs and incubated for 30 min at 37 °C and 5 % CO_2_ to remove excess mucus. Subsequently, PBS was removed and 500 µl of pre-warmed PBS was added to the apical compartment to perform measurements of transepithelial electrical resistance (TEER) using an EVOM3 volt/Ohm meter (World Precision Instruments). For analysis of transepithelial resistance, TEER values were multiplied by the surface area of transwell filters (0.47 cm^2^). E2 passages of H5N1-2020, H5N1-2021 PB2-627E and H5N1-2021 PB2-627K virus isolates were diluted in DMEM Glutamax immediately before inoculation. After aspiration of apical PBS, 10 µl of virus inoculum at a dose of 10^6^ EID_50_ (H5N1-2020 10^4.82^ TCID_50_, H5N1-2021 PB2-627E 10^5.61^ TCID_50_ and H5N1-2021 PB2-627K 10^4.85^ TCID_50_) was pipetted apically onto the WD-AECs and incubated at 37 °C, 5 % CO_2_ for the duration of the experiment. After 4 h, five apical washes with 500 µl pre-warmed PBS were performed and discarded to remove unbound virus, and a sixth wash was collected for subsequent RT-qPCR and virus isolation analysis as t=4 h sample. After 24, 48 and 72 h post-inoculation (hpi), apical washes were collected using 500 µl pre-warmed PBS. On 24 and 72 h, TEER measurements were performed after collection of apical washes. The TEER electrode was thoroughly cleaned between each WD-AEC to avoid cross-contamination. The experiment was terminated at 72 hpi by fixation of the WD-AECs with 4 % paraformaldehyde. Apical washes were stored at −70 °C until further processing.

### Influenza A qRT-PCR

To evaluate viral nucleic acid replication, 200 µl of each apical wash was used for RNA extraction. For standard curves, tenfold dilutions of all three H5N1 virus stocks were prepared in 2.95 % tryptose phosphate broth and stored at −70 °C. Viral RNA was extracted from apical washes and standard curves using the MagNa Pure 96 system (Roche), and viral nucleic acid was amplified using qRT-PCR targeting the matrix gene, as described previously [18]. EID_50_-equivalent titres were calculated using the standard curve.

### Virus titration

Virus titration of apical wash samples on MDCK cells (Philips-Duphar) was performed to determine whether infectious virus was produced on bovine WD-AECs. MDCK cells were maintained in DMEM GlutaMAX supplemented with 5 % fetal calf serum (Capricorn scientific) and 0.1 % penicillin-streptomycin (Thermo Fisher Scientific) at 37 °C and 5 % CO_2_. Endpoint titration was used to determine the TCID_50_ : 3×10^4^ MDCK cells were seeded per well of 96-well plates on the day prior to inoculation with apical wash samples. After two washes with PBS, tenfold serial dilutions of apical washes in DMEM GlutaMAX supplemented with 0.1 % penicillin-streptomycin and 0.3 % bovine serum albumin (BSA) were prepared and added to the monolayers. After 2 days at 37 °C and 5 % CO_2_, monolayers were washed two times with PBS before drying, freezing at −20 °C for ≥1 h and fixation with 4 % paraformaldehyde. The HB 65 mouse anti-NP antibody (0.17 ug ml^−1^, in-house) was used to detect viral antigen, followed by staining with a goat anti-mouse antibody conjugated with AF647 (dilution 1:1000, Thermo Fisher Scientific). Then 1 µg ml^−1^ 4′,6-Diamidine-2′-phenylindole dihydrochloride (DAPI) (Roche) was used as a counterstain. After drying at 37 °C, all wells were imaged using a BioTek Cytation 1 (Agilent) inverted microscope at a magnification of 2.5×, with empirically determined exposure time and imaging autofocus on DAPI stained cells. Subsequently, the number of total cells (DAPI +nuclei) and the number of infected cells (AF647 +cells) were calculated using Gen5 3.11. A well was marked positive if ≥20 spots were counted in the AF647 channel. TCID_50_ was calculated using the Spaerman-Kärber algorithm [19,20].

### Histology and immunohistochemistry

To obtain transverse sections for general morphology, uninfected WD-AECs were fixed in 10 % neutral buffered formalin and embedded in paraffin blocks according to general pathology principles. Seven micrometre sections were cut on a microtome and stained using hematoxylin and eosin. For immunohistochemical detection of p63, heat-induced antigen retrieval was performed for 15 min at 100 °C in 10 mM citate buffer at pH6 (DAKO) in a pressure cooker, while no antigen retrieval was required for detection of acetylated tubulin. Unspecific antigen binding was blocked for 30 min at room temperature in normal goat serum, followed by incubation with either anti-p63 (clone 4A4, dilution 1 : 100, Abcam) or anti-acetylated tubulin (clone 6-11B-1, dilution 1 : 100, Thermo Fisher Scientific) in PBS with 1 % BSA for 45 min at room temperature. Primary antibody detection was performed using the EnVision+System HRP (DAKO) for 30 min, followed by incubation with 3,3'-diaminobenzidine (DAB) for 5 min at room temperature before counterstaining with Mayer’s hematoxylin and mounting with Eukitt mounting medium (Sigma Aldrich).

### Immunofluorescence

WD-AECs were fixed in 4 % paraformaldehyde for 1 h at room temperature. After permeabilization of WD-AECs with 0.25 % Triton X-100 in PBS for 10 min at room temperature, unspecific binding was blocked using 5 % BSA in PBS for 1 h at room temperature. For epithelial cell marker staining, anti-ZO-1 Alexa Fluor 594 (clone ZO1-1A12, dilution 1 : 50, Thermo Fisher Scientific) and anti-Muc5AC FITC (clone 1–13 M1, dilution 1 : 50, Thermo Fisher Scientific) both diluted in 1 % BSA in PBS were incubated overnight at 4 °C. The next day, the first two antibodies were removed and anti-acTub-555 (clone 7E5H8, dilution 1 : 50, Thermo Fisher Scientific) diluted in 1 % BSA in PBS was added for 1 h at room temperature. For staining of influenza virus nucleoprotein, HB 65 anti-NP antibody (dilution 1 : 500, in-house) was incubated for 1 h at room temperature, followed by goat anti-mouse conjugated to Alexa Fluor 647 (dilution 1 : 500, Thermo Fisher Scientific) for 1 h. Finally, nuclei were counterstained using DAPI for 10 min at room temperature before mounting in ProLong Diamond Antifade mountant (Thermo Fisher scientific). Confocal images were obtained using a Leica Stellaris 5 WLL confocal laser scanning microscope. For the infection experiment, two random fields-of-view in four quadrants were imaged for each WD-AEC filter at 40× magnification (total eight fields-of-view per WD-AEC filter). Total number of ciliated cells and goblet cells were counted by making a maximum projection of the z-stack, subtracting the background and thresholding the images. The background subtracted, thresholded ZO-1 staining was used as a mask to identify cell borders. Total number of nuclei and virus positive cells were counted on background subtracted, thresholded images (without ZO-1 mask). Cells were automatically counted using ImageJ version 2. 14.0 (macro available upon request) [25].

## Results

### Genetic distance between European and North American H5N1 lineages

**Fig. 1.**
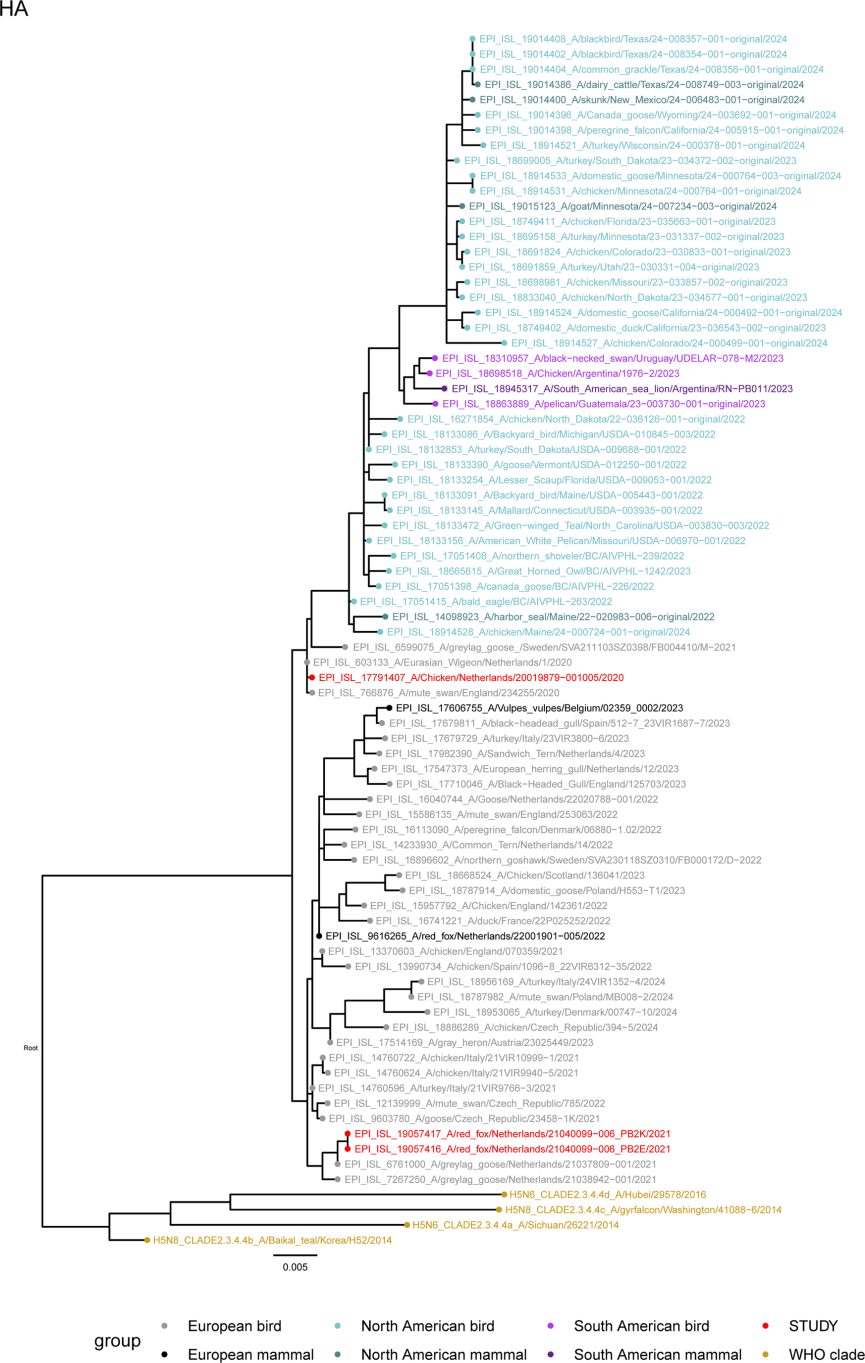
Phylogenetic tree of the influenza hemagglutinin segment with the strains used in this study (red), the WHO subclade 2.3.4.4a, 2.3.4.4b, 2.3.4.4c and 2.3.4.4d representative strains (yellow), the HPAI H5N1 strains isolated in ruminants and a random selection of HPAI strains from the European (EU) (black/grey), North-American (NA) (blue/light blue) and South-American (SA) (purple/light purple) outbreak seasons (2020 to 2024) isolated from birds (light shading) and mammals (dark shading) inferred using maximum likelihood methods. Additional strains were obtained from the GISAID database (see Table S1).

To evaluate the genetic relationship between European and North American H5N1 isolates, phylogenetic analyses were performed. Analysis of the hemagglutinin segment indicated that the HPAI H5N1 viruses isolated from ruminants in North America are closely related to poultry and wild bird viruses isolated in North America (Fig. 1). Mammalian isolates do not cluster separately, which would be expected in case of sustained mammal-to-mammal transmission. Instead, infections of mammals are likely separate transmission events from poultry or wild birds. The North American lineage that includes the ruminant isolates is more closely related to a cluster of sequences detected in South America than to viruses isolated in Europe. Furthermore, the European H5N1-2020 virus used in the current study is more closely related to the North American lineage than the H5N1 virus isolated during the 2021 season, indicating that the North American and European lineage have evolved separately. Other mammalian sequences from Europe are randomly distributed over the cluster of European sequences, similar to the mammalian sequences in North America. Thus, a genetic distance is observed between the European and North American Hemagglutinin segments. A similar pattern is observed for the other seven HPAI segments (Fig. S1).

### Establishment of bovine bronchial epithelial cell-derived air-liquid interface cultures

**Fig. 2.**
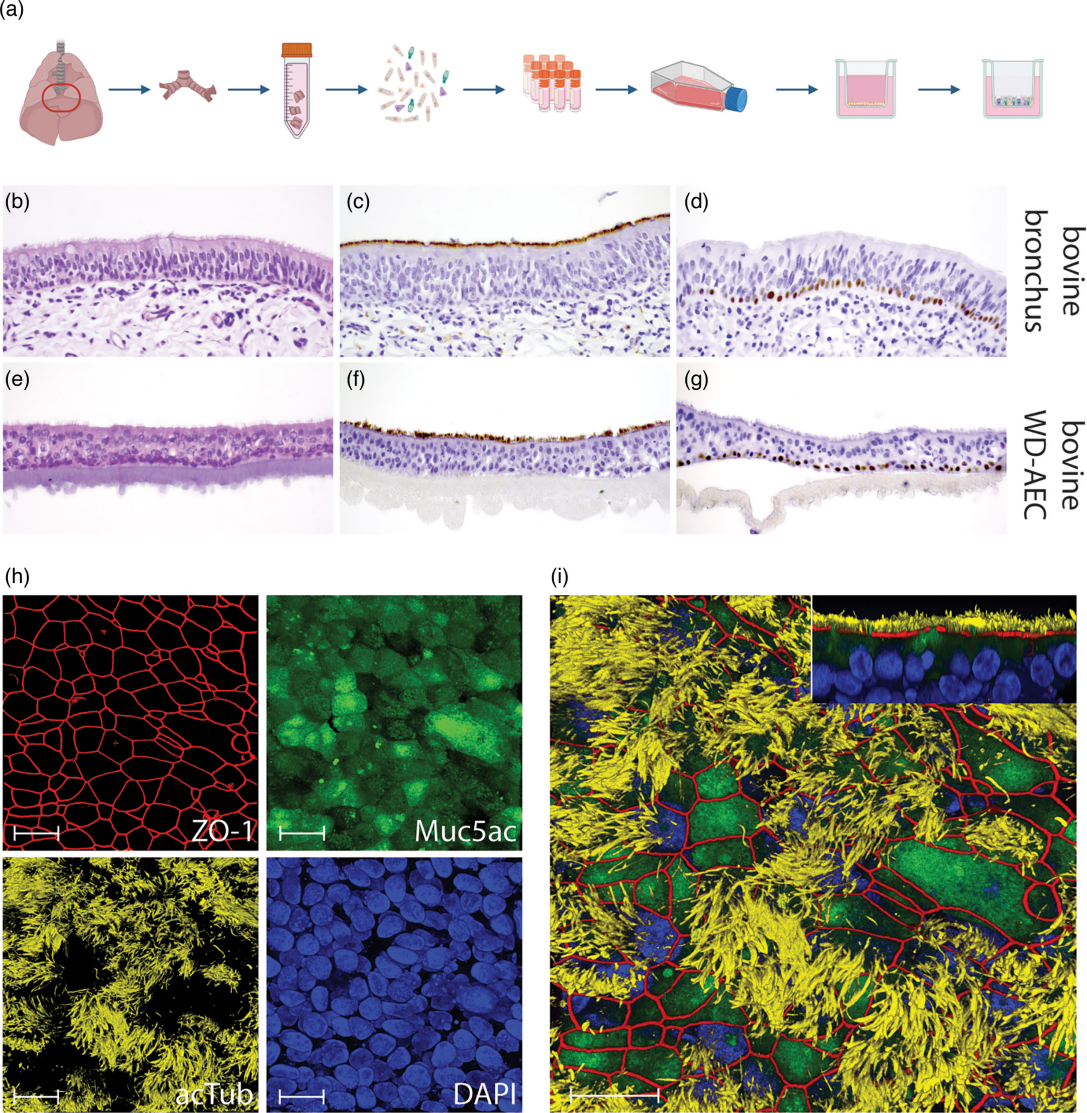
Characterization of bovine WD-AEC. (**a**) Schematic illustration of isolation, expansion, and differentiation of bovine bronchial epithelial cells. (**b**) H and E staining of bronchial epithelium *in vivo,* showing pseudostratified ciliated epithelium, 40× objective. (**c**) Immunohistochemistry (IHC) staining of acetylated tubulin to visualize ciliated cells in bronchial epithelium, 40× objective. (**d**) IHC staining of p63 to visualize basal cells in bronchial epithelium, 40× objective. (**e**) H and E staining of bronchial epithelial cells differentiated at ALI *in vitro,* 40× objective. A pseudostratified ciliated epithelium resembling *in vivo* bronchial epithelium has developed over a 7 weeks differentiation time, although the thickness is decreased as compared to (**b**). (**f**) IHC staining of acetylated tubulin to visualize ciliated cells on WD-AECs, 40× objective. Compared to (**c**), cilia density appears to be slightly reduced. (**g**) IHC staining of p63 to visualize basal cells on WD-AECs, 40× objective. Only basal cells are stained positive, similar as in (**d**). (**h**) Confocal microscopy images, 100× objective, individual channels. ZO-1 staining to visualize tight junctions, Muc5ac staining to visualize goblet cells, acetylated tubulin staining to visualize cilia, DAPI to visualize nuclei. Scalebar=50 µm. (**i**) Overlay of the images shown at (**h**). The insert on the upper right shows a side-view of z-stacks. Scalebar=50 µm.

As a potential bridge between animal experiments and studies conducted in conventional immortalized cell lines, we established bovine airway epithelial-cell derived ALI cultures. Briefly, airway epithelial cells were isolated from primary bronchi of cattle, seeded on transwell filters and differentiated by addition of growth factors and exposure to air (Fig. 2a) [16]. The AEC cultures differentiated over 3–4 weeks into a pseudostratified respiratory epithelium containing basal cells, goblet cells and ciliated cells (Fig. 2e–i and video S1), with an overall similar morphology but decreased thickness compared to the donor tissue (Fig. 2b–d). In our laboratory, WD-AEC cultures remain viable for approximately 12 weeks post-airlift (data not shown). WD-AEC filters were used for subsequent H5N1 infection experiments.

### Bovine well-differentiated airway epithelial cells grown at air-liquid interface are susceptible and permissive to infection with European H5N1 strains

**Fig. 3.**
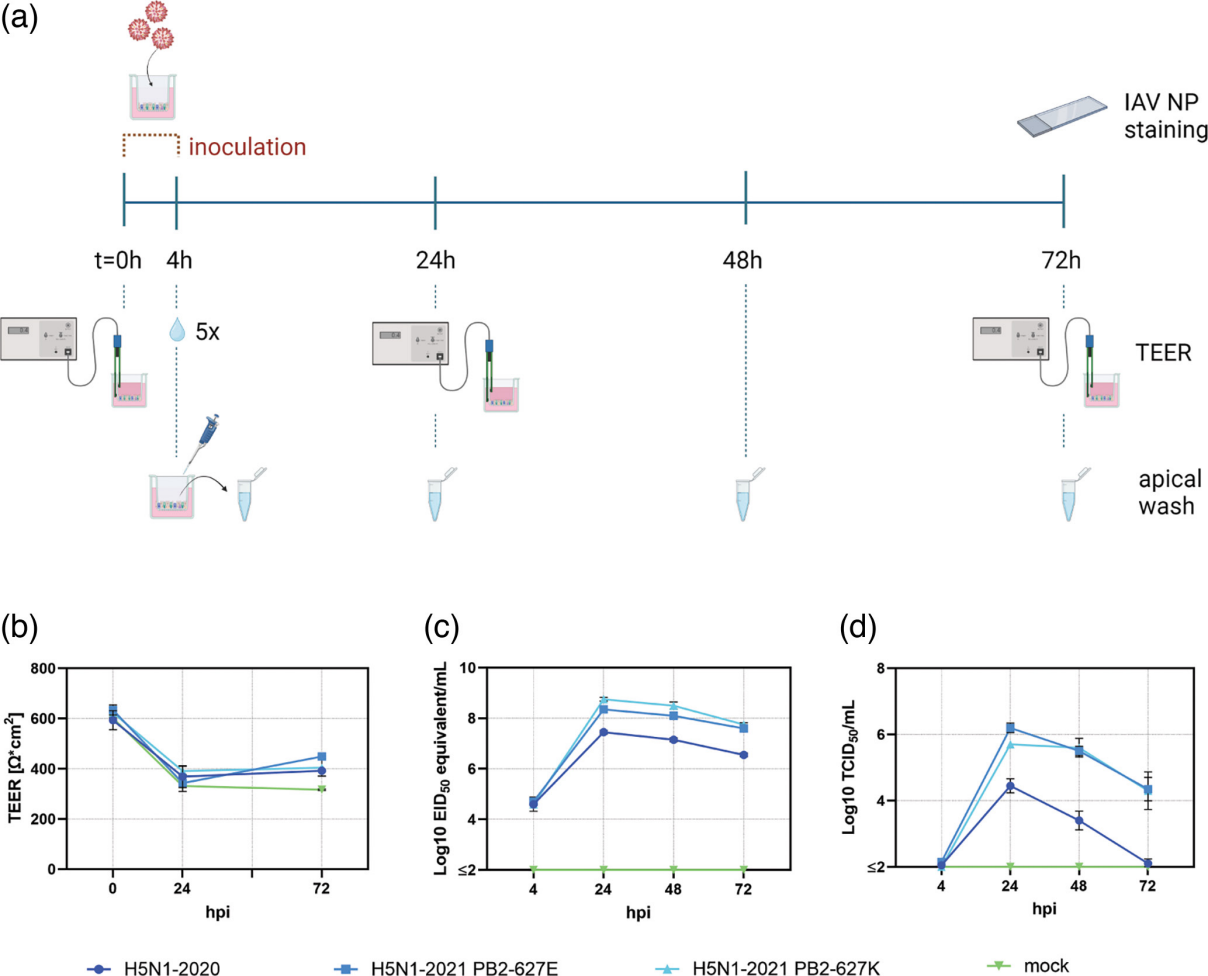
Infection of bovine WD-AECs with H5N1. (**a**) Schematic illustration of the experimental design of HPAI H5N1 inoculation of bovine WD-AECs cultures. A dose of 10^6^ EID_50_ was inoculated in a volume of 10 µl onto bovine WD-AECs, and incubated for 4 h at 37 °C. Subsequently, the apical side of the transwell filters was washed five times to remove unbound inoculum. A sixth wash was collected as apical wash and frozen for later analysis. Apical washes were also collected at 24, 48 and 72 h post-inoculation (hpi). Transepithelial electrical resistance (TEER) was measured at 0 (before inoculation), 24 and 72 hpi, as a measure of tight junction integrity. At the end of the experiment, WD-AECs were fixed and stained for influenza virus nucleoprotein. (**b**) TEER as a measure of epithelial integrity. All WD-AEC filters, including the mock control, show a decrease in TEER values between 0 and 24 hpi, which is likely due to the extensive washing procedure to remove unbound inoculum. Between 24 and 72 hpi, there was no reduction of TEER values in the inoculated filters, indicating that there was no substantial cytopathogenic effect due to H5N1 infection of bovine WD-AEC cultures. (**c**) Viral genome loads detected by RT-qPCR using M segment primers, expressed as EID_50_ equivalents. All three virus strains replicated in bovine WD-AEC cultures. (**d**) Virus isolation from apical washes in MDCK cells. There was an increase of infectious virus titres between t=4 h and t=24 h, and upon 72 hpi, infectious virus titres were reduced for all three strains. For H5N1-2020, lower titres were obtained than for H5N1-2021 PB2-627E and H5N1-2021 PB2-627K.

To evaluate whether European H5N1 isolates can infect epithelial cells in the bovine respiratory tract, we inoculated bovine WD-AECs with H5N1-2020 (isolated from poultry), H5N1-2021 PB2-627E (isolated from a red fox) and H5N1-2021 PB2-627K (isolated from the same red fox, bearing a mutation in PB2 that is known to support replication in mammalian cells) (Fig. 3a) [17]. All three viruses replicated in bovine WD-AECs, as shown by a strong increase in EID_50_ equivalent titres (measured by RT-qPCR) between 4 hpi and 24 hpi (Fig. 3c). Moreover, the highest EID_50_ equivalent titres were observed in the WD-AEC filters inoculated with H5N1-2021 PB2-627K. TEER measurements were employed to measure the integrity of epithelial tight junctions [26], because influenza A virus infections usually cause strong cytopathogenic effects on infected cells. An initial drop of TEER values was observed between 0 hpi and 24 hpi in all WD-AEC filters, which is likely due to the extensive washing procedure to remove the inoculum at 4 hpi. Post inoculation, there was no difference in TEER values between any of the three H5N1 viruses and mock-inoculated filters (Fig. 3b), indicating that the WD-AEC epithelium remained intact. Infectious virus was isolated from apical washes of all WD-AEC filters inoculated with H5N1 viruses, with a strong increase between 4 and 24 hpi and a slow decrease in titres at later time points. Nevertheless, infectious titres were lower in apical washes derived from H5N1-2020-inoculated WD-AECs compared to H5N1-2021 PB2-627E and PB2-627K (Fig. 3d). To investigate if the infection is abortive or if the viruses are effectively replicating, the virus stocks were additionally titrated on MDCK cells to determine the inoculum dose in TCID_50_ (Table S2). The sum of the collected infectious virus particles over the duration of the experiment was calculated and compared to the starting dose. The inoculum and total amount of collected infectious virus particles of the H5N1-2020 infected bovine WD-AEC was both around 10^4.8^, which may indicate abortive infection if no virus particles were removed during the five subsequent washes at 4 hpi and no degradation of infectious virus particles occurred for the duration of the experiment. More likely, most of the inoculum was washed away at 4 hpi (as indicated by the collected sixth wash which was subsequently assessed as the 4 hpi timepoint by PCR and TCID_50_) and the H5N1-2020 virus replicated to a limited extent. The sum of collected infectious virus particles on bovine WD-AEC inoculated with H5N1-2021 PB2-627E or H5N1-2021 PB2-627K was higher than the inoculum, which indicates effective replication regardless if the inoculum was washed away at 4 hpi or if degradation of the virus has occurred. In conclusion, bovine WD-AECs were susceptible to infection with all three investigated European H5N1 isolates and produced infectious virus.

### Detection of influenza A NP antigen in H5N1-inoculated bovine WD-AECs

**Fig. 4.**
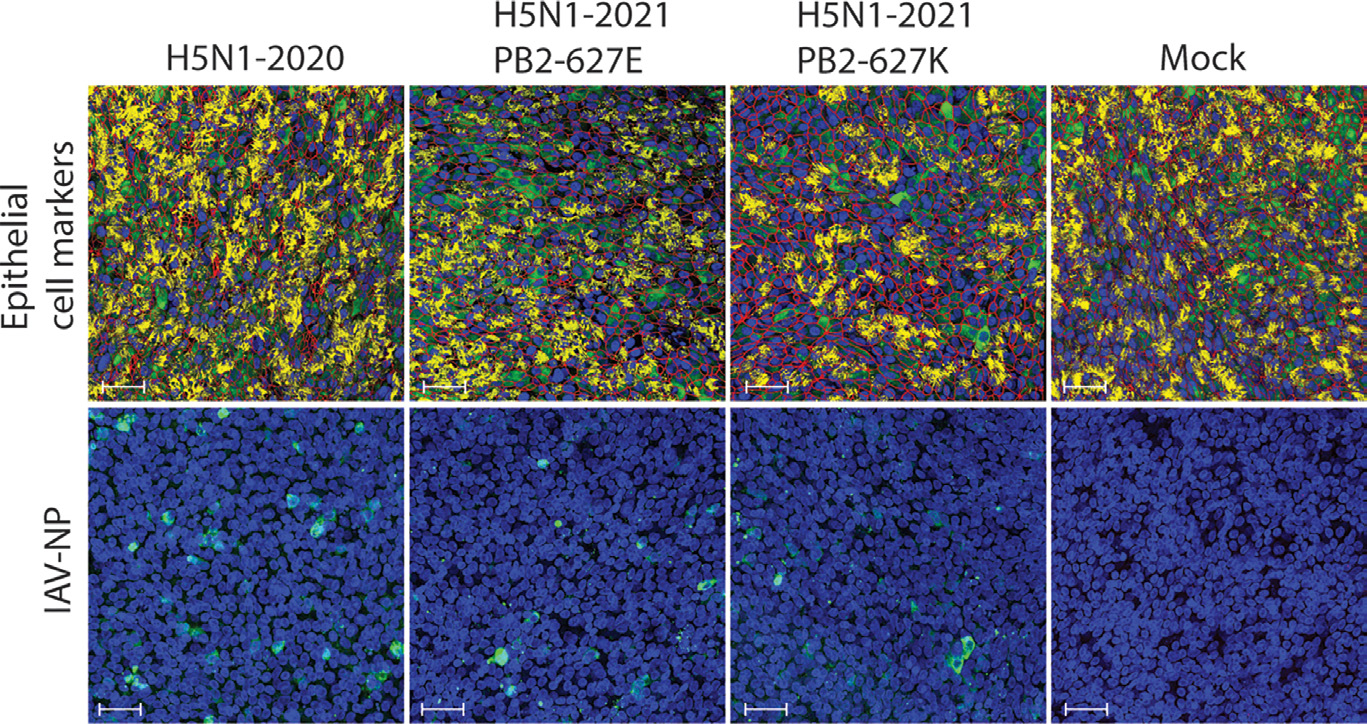
Overlay of bovine WD-AEC cultures infected with three HPAI H5N1 viruses. Upper panels show epithelial cell markers targeting acetylated tubulin (yellow), Muc5AC (green), ZO-1 (red) and nucleic acid (DAPI, blue), indicating the presence of ciliated cells, goblet cells and tight junctions, respectively. Bottom panels show influenza virus nucleoprotein (green) and subsequent counterstain with DAPI (blue), using the duplicate filters of the infection study. Images show one field of view at 40×, scalebar=15 µm (quantification of total number of cells and influenza virus-infected cells of all fields of view is depicted in Table S3). Separate channels are depicted in Fig. S2.

To assess potential cytopathogenic effects of H5N1 infection of bovine WD-AECs, infected filters were fixed 72 hpi and either stained with a panel targeting airway epithelial cell markers (identical to the panel shown in Fig. 2) or with an anti-nucleoprotein antibody (Fig. 4). Staining of the epithelial cell markers showed no significant differences in numbers of any of the investigated cell types compared to the mock-inoculated filters (Figs 4, S2 and Table S3) supporting the maintained TEER values as described above. At the same time, influenza virus-infected cells were still detectable 72 hpi as shown by intra-cellular staining with a monoclonal antibody to the viral NP in WD-AEC filters inoculated with H5N1, and the absence of intracellular NP in the mock-inoculated WD-AEC filter. The mean number of influenza virus-infected cells was slightly higher (24.0) in the H5N1-2020 infected WD-AEC filter than in the H5N1-2021 PB2-627E (6.6) and H5N1-2021 PB2-627K (7.6) WD-AEC filters (Table S3). In summary, despite a slightly higher number of infected cells for H5N1-2020, WD-AEC morphology remained intact and comparable to mock-inoculated filters.

## Discussion

Here we investigated the susceptibility and permissiveness of bovine WD-AECs to infection with three different HPAI H5N1 viruses isolated in the Netherlands from poultry or a red fox, with either the avian PB2-627E or the mammalian PB2-627K adaptation [17]. Until the recent outbreaks of HPAI H5N1 in dairy cattle in the USA, avian influenza was not considered to spread to ruminants [15]. Reports on the clinical signs in cattle indicate rather mild disease in most of the animals [10]. High viral loads were detected in milk, supporting the possibility of cow-to-cow transmission via milk or milking machines [10,11, 13].

The present study demonstrates that infection of bovine epithelial cells is not unique to the North American HPAI H5N1 lineage, as European HPAI H5N1 viruses readily replicated in bovine WD-AECs and generated infectious virus. The cytopathogenic effect of the three HPAI H5N1 viruses on bovine AECs was limited and infectious virus titres dropped rapidly, suggesting that viral propagation was not very efficient. Virus replication was inversely correlated with the number of influenza virus-infected cells, which may indicate death of infected cells after 72 hpi for the H5N1-2021 PB2-627E and H5N1-2021 PB2-627K virus isolates but not the H5N1-2020 virus isolate. Nevertheless, within the first 24 h after inoculation a substantial increase in viral RNA (depicted as EID_50_ equivalents) and infectious titres was observed. The possibility of an abortive infection was disputed for the H5N1-2021 PB2-627E and H5N1-2021 PB2-627K viruses by the fact that the sum of the total number of collected infectious virus particles exceeded the inoculum dosage. For the H5N1-2020 virus the sum of the total number of collected virus particles was similar to the inoculum dose, which may indicate an abortive infection. However, more likely most of the inoculum was washed away during the five subsequent washes at 4 hpi and the H5N1-2020 virus replicated to a limited extent in WD-AEC. In addition, infected cells were still detectable by immunostaining 72 hpi. This is in contrast with an earlier study in which WD-AECs obtained from monkeys, cats, ferrets, dogs, rabbits or pigs were readily infected with influenza virus pH1N1, but infected cells were not detected in bovine or caprine WD-AECs [27].

Interestingly, the two H5N1 viruses isolated from a red fox in 2021 replicated more efficiently in bovine WD-AECs than the poultry isolate from 2020. We can only speculate if this may be related to the fact that these viruses were derived from a mammal, or to the evolutionary trajectory of European HPAI H5N1 strains. However, the mammalian isolates had a similar genetic composition as H5N1 viruses isolated during the 2021 epizootic from poultry and wild birds [17], supporting the likelihood of the latter. The increased genome production of the fox virus isolate with the PB2-627K mutation is not unexpected, as this mutation is widely associated with increased polymerase function in mammalian cells [28,29].

Virus shedding in the respiratory tract of infected cattle was limited during sampling in the field [10], which may suggest the respiratory tract is not the primary replication site of HPAI in cattle. We hypothesize that this may be due to transient viral shedding in the respiratory tract preceding clinical signs in ruminants. Similar observations have been described for other mammals infected with HPAI H5N1, where disease is not observed in acutely infected subjects and overt clinical signs coincide with viral infection of the central nervous system [17,30].

Further investigation will be required to assess potential differences in the capacity of North American or European lineage H5N1 isolates to infect ruminants and if the respiratory tract can act as potential entry site for these viruses. However, the data shown in the current study demonstrate that European H5N1 viruses are able to replicate in bovine airway epithelial cells, creating an opportunity for further viral adaptation to ruminants. At present, surveillance of avian influenza is largely focused on wild birds, poultry and wild carnivorous mammals. However, the H5N1 outbreaks in ruminants in the USA and the results of the current study support extension of serological surveillance to include ruminants. Moreover, it urges to test ruminants showing disease signs as reported in the cases in the USA for highly pathogenic avian influenza infection. Detection of high viral loads in the milk of infected cows poses a potential risk for farmers or consumers of unpasteurized milk. Further investigation into the tropism, shedding and clinical symptoms of the recent HPAI H5N1 virus is required to provide an informed risk assessment for ruminants and the possible implications this may have for the zoonotic potential of HPAI H5N1 viruses.

This study has several limitations. The results cannot predict if the European HPAI H5N1 viruses may have spread to ruminants, or if transmission between ruminants is possible. However, this study shows first indications of amplification of infectious virus particles in bovine WD-AECs, with limited tissue damage, suggesting that subclinical infections may have occurred in cattle during the 2020–2024 European H5N1 epizootics. Further comparison of North American and European HPAI H5N1 virus isolates in cells and tissues of ruminants and more extensive biological replicates are required to assess the translational value of our results. Screening cattle populations, that may have been exposed to HPAI infected birds, for avian influenza virus antibodies is indicated to confirm if these kind of infections have also occurred in Europe.

Our study concludes that European HPAI H5N1 isolates can replicate in bovine WD-AECs, refuting the general assumption that ruminants are not susceptible to infection with influenza A viruses. Our study underscores the benefit of WD-AEC cultures for the pandemic preparedness toolkit, providing a rapid assessment of the host range of an emerging pathogen.

## supplementary material

10.1099/jgv.0.002007 Supplementary Material 1.

10.1099/jgv.0.002007 video 1.

## Abbreviations

AECBM: airway epithelial cell basal medium
AECDM: airway epithelial cell differentiation medium
ALI: air-liquid interface
BPE: bovine pituitary extract
BSA: bovine serum albumin
EID50: 50% egg infectious dose
hEGF: human epidermal growth factor
HPAI: highly pathogenic avian influenza
hpi: hours post-inoculation
IHC: immunohistochemistry
MDCK: Madin-Darby canine kidney
SPF: specific pathogen free
TCID50: 50% tissue culture infectious dose
TEER: trans-epithelial elctrical resistance
WD-AEC: well-differentiated airway epithelial cells

## References

[1] Guan Y, Peiris M, Kong KF, Dyrting KC, Ellis TM (2002). H5N1 influenza viruses isolated from geese in Southeastern China: evidence for genetic reassortment and interspecies transmission to ducks. Virology.

[2] Verhagen JH, Fouchier RAM, Lewis N (2021). Highly pathogenic avian influenza viruses at the wild-domestic bird interface in Europe: future directions for research and surveillance. Viruses.

[3] Cattoli G, Fusaro A, Monne I, Capua I (2009). H5N1 virus evolution in Europe-an updated overview. Viruses.

[4] Adlhoch C, Fusaro A, Gonzales JL, EFSA, ECDC (2020). Avian influenza overview August–December 2020. EFSA J.

[5] Adlhoch C, Fusaro A, EFSA, ECDC, EURL (2022). Scientific report: avian influenza overview June–September 2022. EFSA J.

[6] Caliendo V, Lewis NS, Pohlmann A, Baillie SR, Banyard AC (2022). Transatlantic spread of highly pathogenic avian influenza H5N1 by wild birds from Europe to North America in 2021. Sci Rep.

[7] Charostad J, Rezaei Zadeh Rukerd M, Mahmoudvand S, Bashash D, Hashemi SMA (2023). A comprehensive review of highly pathogenic avian influenza (HPAI) H5N1: an imminent threat at doorstep. Travel Med Infect Dis.

[8] USDA Detections of highly pathogenic avian influenza. https://www.aphis.usda.gov/livestock-poultry-disease/avian/avian-influenza/hpai-detections.

[9] AVMA Goat in Minnesota tests positive for HPAI. https://www.avma.org/news/goat-minnesota-tests-positive-hpai.

[10] USDA Detection of highly pathogenic avian influenza (H5N1) in dairy herds: frequently asked questions. https://www.aphis.usda.gov/livestock-poultry-disease/avian/avian-influenza/hpai-detections/livestock.

[11] Nguyen T-Q, Hutter C, Markin A, Thomas M, Lantz K Emergence and interstate spread of highly pathogenic avian influenza A(H5N1) in dairy cattle. bioRxiv.

[12] CDC Highly pathogenic avian influenza A (H5N1) virus infection reported in a person in the U.S. https://www.cdc.gov/media/releases/2024/p0401-avian-flu.html.

[13] CDC Technical update: summary analysis of genetic sequences of highly pathogenic avian influenza A(H5N1) viruses in Texas. https://www.cdc.gov/flu/avianflu/spotlights/2023-2024/h5n1-analysis-texas.htm.

[14] Zhang X, Xu G, Wang C, Jiang M, Gao W (2017). Enhanced pathogenicity and neurotropism of mouse-adapted H10N7 influenza virus are mediated by novel PB2 and NA mutations. J Gen Virol.

[15] Sreenivasan CC, Thomas M, Kaushik RS, Wang D, Li F (2019). Influenza A in Bovine species: a narrative literature review. Viruses.

[16] Gultom M, Laloli L, Dijkman R Coronaviruses: Methods and Protocols.

[17] Bordes L, Vreman S, Heutink R, Roose M, Venema S (2023). Highly Pathogenic Avian Influenza H5N1 Virus Infections in Wild Red Foxes (Vulpes vulpes) Show Neurotropism and Adaptive Virus Mutations. Microbiol Spectr.

[18] Bouwstra R, Koch G, Heutink R, Harders F, Spek A (2014). Phylogenetic analysis of highly pathogenic avian influenza A (H5N8) virus outbreak strains provides evidence for four separate introductions and one between-poultry farm transmission in the. Eurosurveillance.

[19] Spearman C (1908). The method of right and wrong cases (constant stimuli) without Gauss’s formulae. Br J Psychol.

[20] Kärber G (1931). Beitrag zur kollektiven Behandlung pharmakologischer Reihenversuche. Naunyn-Schmiedebergs Archiv für experimentelle pathologie und pharmakologie.

[21] Katoh K, Toh H (2010). Parallelization of the MAFFT multiple sequence alignment program. Bioinformatics.

[22] Nguyen L-T, Schmidt HA, von Haeseler A, Minh BQ (2015). IQ-TREE: a fast and effective stochastic algorithm for estimating maximum-likelihood phylogenies. Mol Biol Evol.

[23] Yu GC, Smith DK, Zhu HC, Guan Y, Lam TTY (2017). GGTREE: an R package for visualization and annotation of phylogenetic trees with their covariates and other associated data. Methods Ecol Evol.

[24] Shu YL, McCauley J (2017). GISAID: global initiative on sharing all influenza data - from vision to reality. Euro Surveill.

[25] Schindelin J, Arganda-Carreras I, Frise E, Kaynig V, Longair M (2012). Fiji: an open-source platform for biological-image analysis. Nat Methods.

[26] Srinivasan B, Kolli AR, Esch MB, Abaci HE, Shuler ML (2015). TEER measurement techniques for in vitro barrier model systems. J Lab Autom.

[27] Gultom M, Licheri M, Laloli L, Wider M, Strässle M (2021). Susceptibility of well-differentiated airway epithelial cell cultures from domestic and wild animals to severe acute respiratory syndrome coronavirus 2. Emerg Infect Dis.

[28] Subbarao EK, London W, Murphy BR (1993). A single amino acid in the PB2 gene of influenza A virus is a determinant of host range. J Virol.

[29] Steel J, Lowen AC, Mubareka S, Palese P (2009). Transmission of influenza virus in a mammalian host is increased by PB2 amino acids 627K or 627E/701N. PLoS Pathog.

[30] Vreman S, Kik M, Germeraad E, Heutink R, Harders F (2023). Zoonotic mutation of highly pathogenic avian influenza H5N1 virus identified in the brain of multiple wild carnivore species. Pathogens.

