# Supplementary material for: H5N1 clade 2.3.4.4b avian influenza viruses replicate in differentiated bovine airway epithelial cells cultured at air-liquid interface
Source: J Gen Virol. 2024 Jun 26;105(6):002007. doi: 10.1099/jgv.0.002007 (PMC11256440; doi:10.1099/jgv.0.002007)
Supplement: Supplementary Material 1. [file jgv-105-02007-s001.pdf]

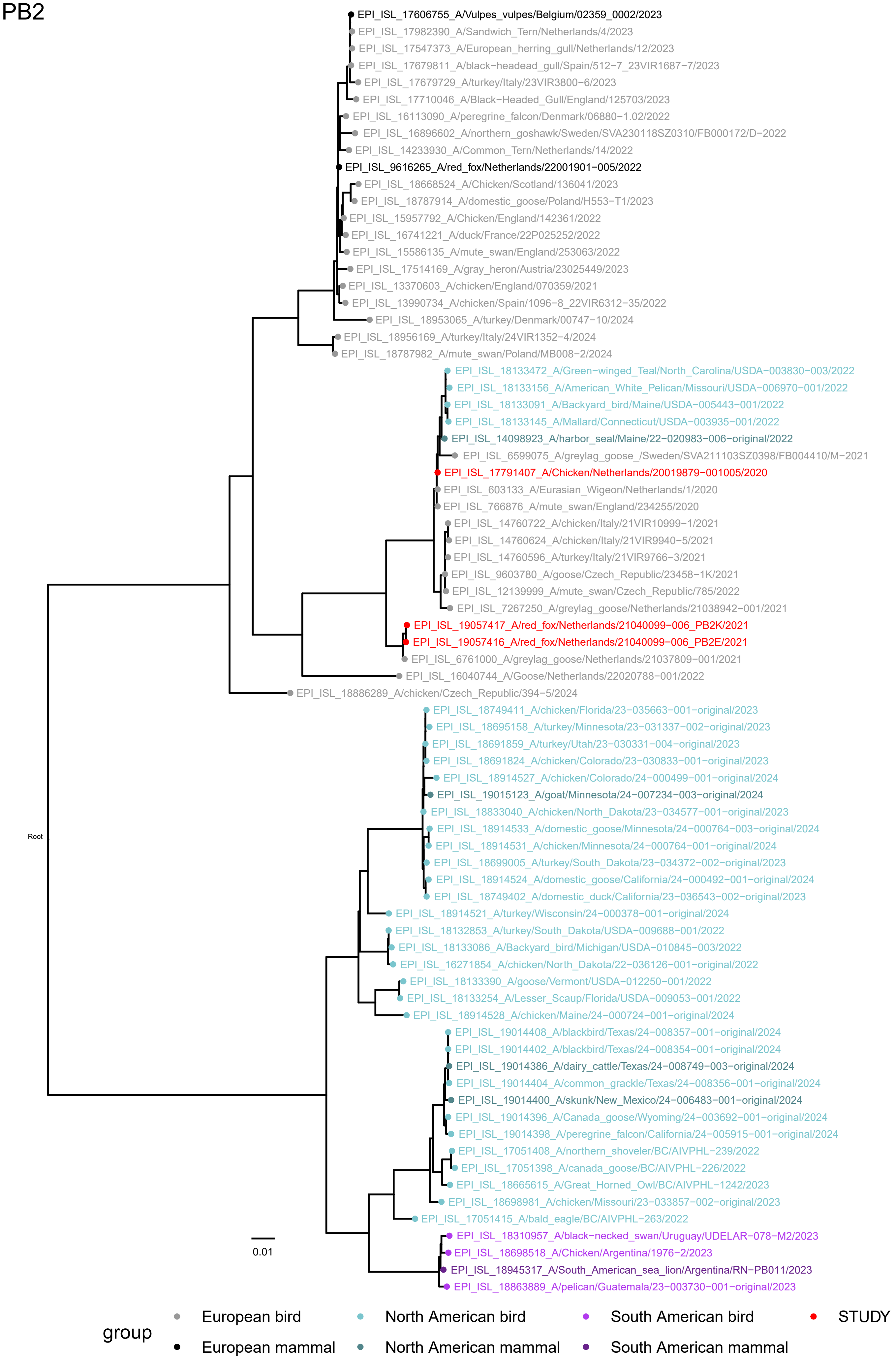

Supplementary figure 1

Phylogenetic tree of all segments (except HA) with the virus sequences used in this study (red), the HPAI H5N1 strains isolated in ruminants and a random selection of HPAI strains from the European (EU)(black/grey), North-American (NA)(blue/light blue) and South-American (SA)(purple/light purple) outbreak seasons (2020 to 2024) isolated from birds (light shading) and mammals (dark shading) inferred using maximum likelihood (ML) methods. Additional strains were obtained from the GISAID database (see supplementary table 1).

PB1

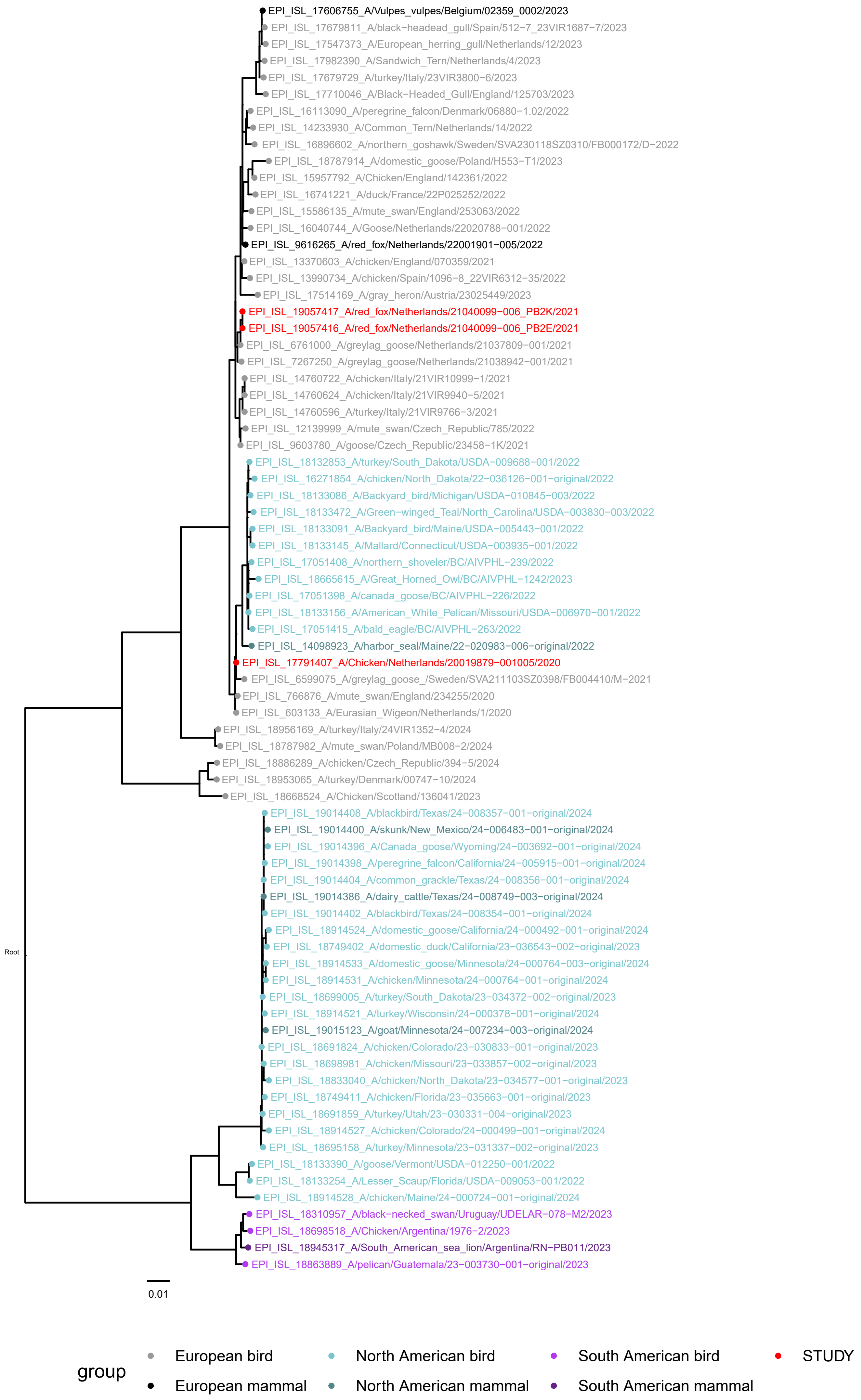
EPI_ISL_17606755_A/Vulpes_vulpes/Belgium/02359_0002/2023
EPI_ISL_17679811_A/black−headead_gull/Spain/512−7_23VIR1687−7/2023
EPI_ISL_17547373_A/European_herring_gull/Netherlands/12/2023
EPI_ISL_17982390_A/Sandwich_Tern/Netherlands/4/2023
EPI_ISL_17679729_A/turkey/Italy/23VIR3800−6/2023
EPI_ISL_17710046_A/Black−Headed_Gull/England/125703/2023
EPI_ISL_16113090_A/peregrine_falcon/Denmark/06880−1.02/2022
EPI_ISL_14233930_A/Common_Tern/Netherlands/14/2022
EPI_ISL_16896602_A/northern_goshawk/Sweden/SVA230118SZ0310/FB000172/D−2022
EPI_ISL_18787914_A/domestic_goose/Poland/H553−T1/2023
EPI_ISL_15957792_A/Chicken/England/142361/2022
EPI_ISL_16741221_A/duck/France/22P025252/2022
EPI_ISL_15586135_A/mute_swan/England/253063/2022
EPI_ISL_16040744_A/Goose/Netherlands/22020788−001/2022
EPI_ISL_9616265_A/red_fox/Netherlands/22001901−005/2022
EPI_ISL_13370603_A/chicken/England/070359/2021
EPI_ISL_13990734_A/chicken/Spain/1096−8_22VIR6312−35/2022
EPI_ISL_17514169_A/gray_heron/Austria/23025449/2023
EPI_ISL_19057417_A/red_fox/Netherlands/21040099−006_PB2K/2021
EPI_ISL_19057416_A/red_fox/Netherlands/21040099−006_PB2E/2021
EPI_ISL_6761000_A/greylag_goose/Netherlands/21037809−001/2021
EPI_ISL_7267250_A/greylag_goose/Netherlands/21038942−001/2021
EPI_ISL_14760722_A/chicken/Italy/21VIR10999−1/2021
EPI_ISL_14760624_A/chicken/Italy/21VIR9940−5/2021
EPI_ISL_14760596_A/turkey/Italy/21VIR9766−3/2021
EPI_ISL_12139999_A/mute_swan/Czech_Republic/785/2022
EPI_ISL_9603780_A/goose/Czech_Republic/23458−1K/2021
EPI_ISL_18132853_A/turkey/South_Dakota/USDA−009688−001/2022
EPI_ISL_16271854_A/chicken/North_Dakota/22−036126−001−original/2022
EPI_ISL_18133086_A/Backyard_bird/Michigan/USDA−010845−003/2022
EPI_ISL_18133472_A/Green−winged_Teal/North_Carolina/USDA−003830−003/2022
EPI_ISL_18133091_A/Backyard_bird/Maine/USDA−005443−001/2022
EPI_ISL_18133145_A/Mallard/Connecticut/USDA−003935−001/2022
EPI_ISL_17051408_A/northern_shoveler/BC/AIVPHL−239/2022
EPI_ISL_18665615_A/Great_Horned_Owl/BC/AIVPHL−1242/2023
EPI_ISL_17051398_A/canada_goose/BC/AIVPHL−226/2022
EPI_ISL_18133156_A/American_White_Pelican/Missouri/USDA−006970−001/2022
EPI_ISL_17051415_A/bald_eagle/BC/AIVPHL−263/2022
EPI_ISL_14098923_A/harbor_seal/Maine/22−020983−006−original/2022
EPI_ISL_17791407_A/Chicken/Netherlands/20019879−001005/2020
EPI_ISL_6599075_A/greylag_goose_/Sweden/SVA211103SZ0398/FB004410/M−2021
EPI_ISL_766876_A/mute_swan/England/234255/2020
EPI_ISL_603133_A/Eurasian_Wigeon/Netherlands/1/2020
EPI_ISL_18956169_A/turkey/Italy/24VIR1352−4/2024
EPI_ISL_18787982_A/mute_swan/Poland/MB008−2/2024
EPI_ISL_18886289_A/chicken/Czech_Republic/394−5/2024
EPI_ISL_18953065_A/turkey/Denmark/00747−10/2024
EPI_ISL_18668524_A/Chicken/Scotland/136041/2023
EPI_ISL_19014408_A/blackbird/Texas/24−008357−001−original/2024
EPI_ISL_19014400_A/skunk/New_Mexico/24−006483−001−original/2024
EPI_ISL_19014396_A/Canada_goose/Wyoming/24−003692−001−original/2024
EPI_ISL_19014398_A/peregrine_falcon/California/24−005915−001−original/2024
EPI_ISL_19014404_A/common_grackle/Texas/24−008356−001−original/2024
EPI_ISL_19014386_A/dairy_cattle/Texas/24−008749−003−original/2024
EPI_ISL_19014402_A/blackbird/Texas/24−008354−001−original/2024
EPI_ISL_18914524_A/domestic_goose/California/24−000492−001−original/2024
EPI_ISL_18749402_A/domestic_duck/California/23−036543−002−original/2023
EPI_ISL_18914533_A/domestic_goose/Minnesota/24−000764−003−original/2024
EPI_ISL_18914531_A/chicken/Minnesota/24−000764−001−original/2024
EPI_ISL_18699005_A/turkey/South_Dakota/23−034372−002−original/2023
EPI_ISL_18914521_A/turkey/Wisconsin/24−000378−001−original/2024
EPI_ISL_19015123_A/goat/Minnesota/24−007234−003−original/2024
EPI_ISL_18691824_A/chicken/Colorado/23−030833−001−original/2023
EPI_ISL_18698981_A/chicken/Missouri/23−033857−002−original/2023
EPI_ISL_18833040_A/chicken/North_Dakota/23−034577−001−original/2023
EPI_ISL_18749411_A/chicken/Florida/23−035663−001−original/2023
EPI_ISL_18691859_A/turkey/Utah/23−030331−004−original/2023
EPI_ISL_18914527_A/chicken/Colorado/24−000499−001−original/2024
EPI_ISL_18695158_A/turkey/Minnesota/23−031337−002−original/2023
EPI_ISL_18133390_A/goose/Vermont/USDA−012250−001/2022
EPI_ISL_18133254_A/Lesser_Scaup/Florida/USDA−009053−001/2022
EPI_ISL_18914528_A/chicken/Maine/24−000724−001−original/2024
EPI_ISL_18310957_A/black−necked_swan/Uruguay/UDELAR−078−M2/2023
EPI_ISL_18698518_A/Chicken/Argentina/1976−2/2023
EPI_ISL_18945317_A/South_American_sea_lion/Argentina/RN−PB011/2023
EPI_ISL_18863889_A/pelican/Guatemala/23−003730−001−original/2023
Root
0.01
group
European bird
European mammal
North American bird
North American mammal
South American bird
South American mammal
STUDY

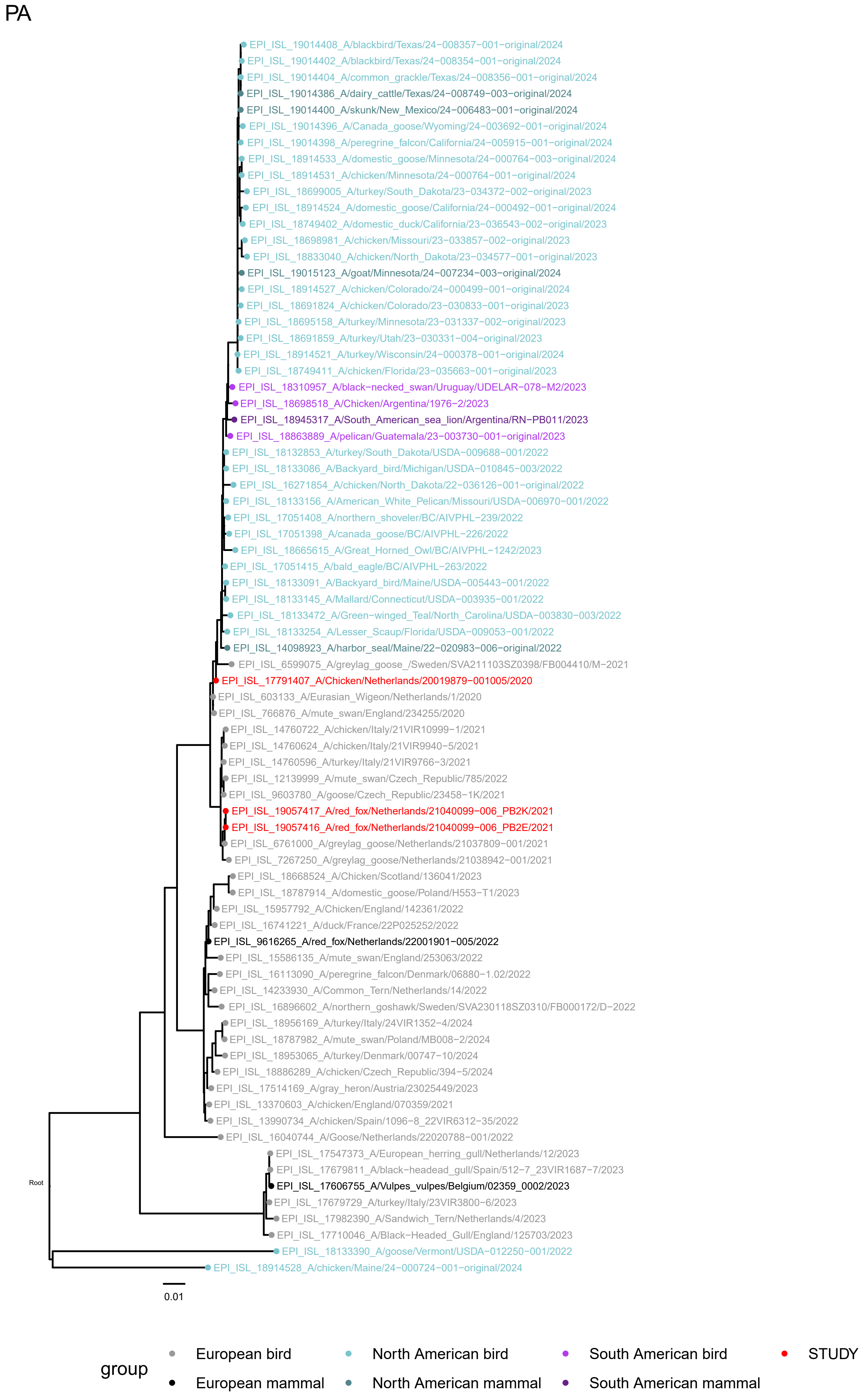
PA
EPI_ISL_19014408_A/blackbird/Texas/24−008357−001−original/2024
EPI_ISL_19014402_A/blackbird/Texas/24−008354−001−original/2024
EPI_ISL_19014404_A/common_grackle/Texas/24−008356−001−original/2024
EPI_ISL_19014386_A/dairy_cattle/Texas/24−008749−003−original/2024
EPI_ISL_19014400_A/skunk/New_Mexico/24−006483−001−original/2024
EPI_ISL_19014396_A/Canada_goose/Wyoming/24−003692−001−original/2024
EPI_ISL_19014398_A/peregrine_falcon/California/24−005915−001−original/2024
EPI_ISL_18914533_A/domestic_goose/Minnesota/24−000764−003−original/2024
EPI_ISL_18914531_A/chicken/Minnesota/24−000764−001−original/2024
EPI_ISL_18699005_A/turkey/South_Dakota/23−034372−002−original/2023
EPI_ISL_18914524_A/domestic_goose/California/24−000492−001−original/2024
EPI_ISL_18749402_A/domestic_duck/California/23−036543−002−original/2023
EPI_ISL_18698981_A/chicken/Missouri/23−033857−002−original/2023
EPI_ISL_18833040_A/chicken/North_Dakota/23−034577−001−original/2023
EPI_ISL_19015123_A/goat/Minnesota/24−007234−003−original/2024
EPI_ISL_18914527_A/chicken/Colorado/24−000499−001−original/2024
EPI_ISL_18691824_A/chicken/Colorado/23−030833−001−original/2023
EPI_ISL_18695158_A/turkey/Minnesota/23−031337−002−original/2023
EPI_ISL_18691859_A/turkey/Utah/23−030331−004−original/2023
EPI_ISL_18914521_A/turkey/Wisconsin/24−000378−001−original/2024
EPI_ISL_18749411_A/chicken/Florida/23−035663−001−original/2023
EPI_ISL_18310957_A/black−necked_swan/Uruguay/UDELAR−078−M2/2023
EPI_ISL_18698518_A/Chicken/Argentina/1976−2/2023
EPI_ISL_18945317_A/South_American_sea_lion/Argentina/RN−PB011/2023
EPI_ISL_18863889_A/pelican/Guatemala/23−003730−001−original/2023
EPI_ISL_18132853_A/turkey/South_Dakota/USDA−009688−001/2022
EPI_ISL_18133086_A/Backyard_bird/Michigan/USDA−010845−003/2022
EPI_ISL_16271854_A/chicken/North_Dakota/22−036126−001−original/2022
EPI_ISL_18133156_A/American_White_Pelican/Missouri/USDA−006970−001/2022
EPI_ISL_17051408_A/northern_shoveler/BC/AIVPHL−239/2022
EPI_ISL_17051398_A/canada_goose/BC/AIVPHL−226/2022
EPI_ISL_18665615_A/Great_Horned_Owl/BC/AIVPHL−1242/2023
EPI_ISL_17051415_A/bald_eagle/BC/AIVPHL−263/2022
EPI_ISL_18133091_A/Backyard_bird/Maine/USDA−005443−001/2022
EPI_ISL_18133145_A/Mallard/Connecticut/USDA−003935−001/2022
EPI_ISL_18133472_A/Green−winged_Teal/North_Carolina/USDA−003830−003/2022
EPI_ISL_18133254_A/Lesser_Scaup/Florida/USDA−009053−001/2022
EPI_ISL_14098923_A/harbor_seal/Maine/22−020983−006−original/2022
EPI_ISL_6599075_A/greylag_goose_/Sweden/SVA211103SZ0398/FB004410/M−2021
EPI_ISL_17791407_A/Chicken/Netherlands/20019879−001005/2020
EPI_ISL_603133_A/Eurasian_Wigeon/Netherlands/1/2020
EPI_ISL_766876_A/mute_swan/England/234255/2020
EPI_ISL_14760722_A/chicken/Italy/21VIR10999−1/2021
EPI_ISL_14760624_A/chicken/Italy/21VIR9940−5/2021
EPI_ISL_14760596_A/turkey/Italy/21VIR9766−3/2021
EPI_ISL_12139999_A/mute_swan/Czech_Republic/785/2022
EPI_ISL_9603780_A/goose/Czech_Republic/23458−1K/2021
EPI_ISL_19057417_A/red_fox/Netherlands/21040099−006_PB2K/2021
EPI_ISL_19057416_A/red_fox/Netherlands/21040099−006_PB2E/2021
EPI_ISL_6761000_A/greylag_goose/Netherlands/21037809−001/2021
EPI_ISL_7267250_A/greylag_goose/Netherlands/21038942−001/2021
EPI_ISL_18668524_A/Chicken/Scotland/136041/2023
EPI_ISL_18787914_A/domestic_goose/Poland/H553−T1/2023
EPI_ISL_15957792_A/Chicken/England/142361/2022
EPI_ISL_16741221_A/duck/France/22P025252/2022
EPI_ISL_9616265_A/red_fox/Netherlands/22001901−005/2022
EPI_ISL_15586135_A/mute_swan/England/253063/2022
EPI_ISL_16113090_A/peregrine_falcon/Denmark/06880−1.02/2022
EPI_ISL_14233930_A/Common_Tern/Netherlands/14/2022
EPI_ISL_16896602_A/northern_goshawk/Sweden/SVA230118SZ0310/FB000172/D−2022
EPI_ISL_18956169_A/turkey/Italy/24VIR1352−4/2024
EPI_ISL_18787982_A/mute_swan/Poland/MB008−2/2024
EPI_ISL_18953065_A/turkey/Denmark/00747−10/2024
EPI_ISL_18886289_A/chicken/Czech_Republic/394−5/2024
EPI_ISL_17514169_A/gray_heron/Austria/23025449/2023
EPI_ISL_13370603_A/chicken/England/070359/2021
EPI_ISL_13990734_A/chicken/Spain/1096−8_22VIR6312−35/2022
EPI_ISL_16040744_A/Goose/Netherlands/22020788−001/2022
EPI_ISL_17547373_A/European_herring_gull/Netherlands/12/2023
EPI_ISL_17679811_A/black−headead_gull/Spain/512−7_23VIR1687−7/2023
EPI_ISL_17606755_A/Vulpes_vulpes/Belgium/02359_0002/2023
EPI_ISL_17679729_A/turkey/Italy/23VIR3800−6/2023
EPI_ISL_17982390_A/Sandwich_Tern/Netherlands/4/2023
EPI_ISL_17710046_A/Black−Headed_Gull/England/125703/2023
EPI_ISL_18133390_A/goose/Vermont/USDA−012250−001/2022
EPI_ISL_18914528_A/chicken/Maine/24−000724−001−original/2024
Root
0.01
group
European bird
European mammal
North American bird
North American mammal
South American bird
South American mammal
STUDY

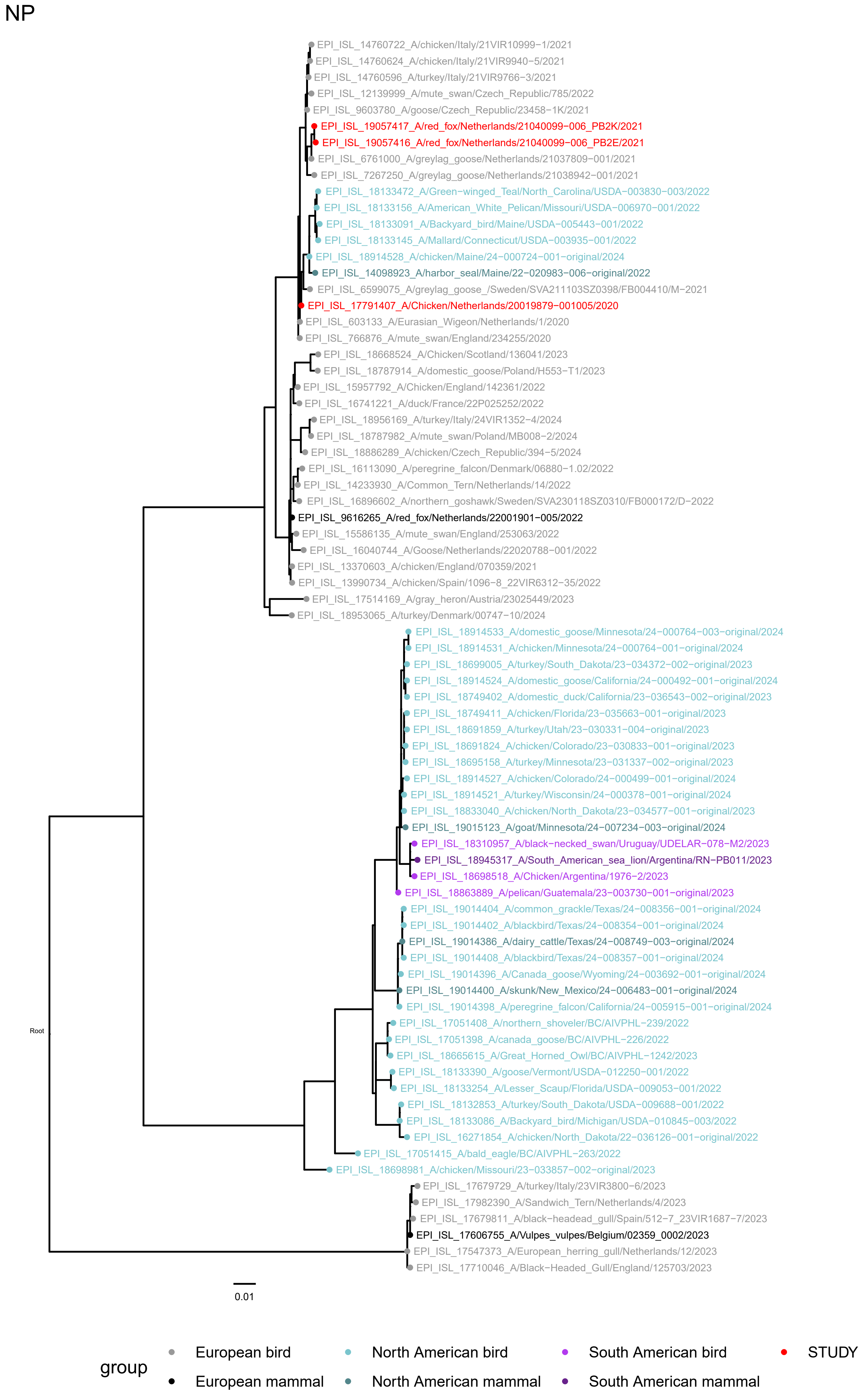
NP
EPI_ISL_14760722_A/chicken/Italy/21VIR10999−1/2021
EPI_ISL_14760624_A/chicken/Italy/21VIR9940−5/2021
EPI_ISL_14760596_A/turkey/Italy/21VIR9766−3/2021
EPI_ISL_12139999_A/mute_swan/Czech_Republic/785/2022
EPI_ISL_9603780_A/goose/Czech_Republic/23458−1K/2021
EPI_ISL_19057417_A/red_fox/Netherlands/21040099−006_PB2K/2021
EPI_ISL_19057416_A/red_fox/Netherlands/21040099−006_PB2E/2021
EPI_ISL_6761000_A/greylag_goose/Netherlands/21037809−001/2021
EPI_ISL_7267250_A/greylag_goose/Netherlands/21038942−001/2021
EPI_ISL_18133472_A/Green−winged_Teal/North_Carolina/USDA−003830−003/2022
EPI_ISL_18133156_A/American_White_Pelican/Missouri/USDA−006970−001/2022
EPI_ISL_18133091_A/Backyard_bird/Maine/USDA−005443−001/2022
EPI_ISL_18133145_A/Mallard/Connecticut/USDA−003935−001/2022
EPI_ISL_18914528_A/chicken/Maine/24−000724−001−original/2024
EPI_ISL_14098923_A/harbor_seal/Maine/22−020983−006−original/2022
EPI_ISL_6599075_A/greylag_goose_/Sweden/SVA211103SZ0398/FB004410/M−2021
EPI_ISL_17791407_A/Chicken/Netherlands/20019879−001005/2020
EPI_ISL_603133_A/Eurasian_Wigeon/Netherlands/1/2020
EPI_ISL_766876_A/mute_swan/England/234255/2020
EPI_ISL_18668524_A/Chicken/Scotland/136041/2023
EPI_ISL_18787914_A/domestic_goose/Poland/H553−T1/2023
EPI_ISL_15957792_A/Chicken/England/142361/2022
EPI_ISL_16741221_A/duck/France/22P025252/2022
EPI_ISL_18956169_A/turkey/Italy/24VIR1352−4/2024
EPI_ISL_18787982_A/mute_swan/Poland/MB008−2/2024
EPI_ISL_18886289_A/chicken/Czech_Republic/394−5/2024
EPI_ISL_16113090_A/peregrine_falcon/Denmark/06880−1.02/2022
EPI_ISL_14233930_A/Common_Tern/Netherlands/14/2022
EPI_ISL_16896602_A/northern_goshawk/Sweden/SVA230118SZ0310/FB000172/D−2022
EPI_ISL_9616265_A/red_fox/Netherlands/22001901−005/2022
EPI_ISL_15586135_A/mute_swan/England/253063/2022
EPI_ISL_16040744_A/Goose/Netherlands/22020788−001/2022
EPI_ISL_13370603_A/chicken/England/070359/2021
EPI_ISL_13990734_A/chicken/Spain/1096−8_22VIR6312−35/2022
EPI_ISL_17514169_A/gray_heron/Austria/23025449/2023
EPI_ISL_18953065_A/turkey/Denmark/00747−10/2024
EPI_ISL_18914533_A/domestic_goose/Minnesota/24−000764−003−original/2024
EPI_ISL_18914531_A/chicken/Minnesota/24−000764−001−original/2024
EPI_ISL_18699005_A/turkey/South_Dakota/23−034372−002−original/2023
EPI_ISL_18914524_A/domestic_goose/California/24−000492−001−original/2024
EPI_ISL_18749402_A/domestic_duck/California/23−036543−002−original/2023
EPI_ISL_18749411_A/chicken/Florida/23−035663−001−original/2023
EPI_ISL_18691859_A/turkey/Utah/23−030331−004−original/2023
EPI_ISL_18691824_A/chicken/Colorado/23−030833−001−original/2023
EPI_ISL_18695158_A/turkey/Minnesota/23−031337−002−original/2023
EPI_ISL_18914527_A/chicken/Colorado/24−000499−001−original/2024
EPI_ISL_18914521_A/turkey/Wisconsin/24−000378−001−original/2024
EPI_ISL_18833040_A/chicken/North_Dakota/23−034577−001−original/2023
EPI_ISL_19015123_A/goat/Minnesota/24−007234−003−original/2024
EPI_ISL_18310957_A/black−necked_swan/Uruguay/UDELAR−078−M2/2023
EPI_ISL_18945317_A/South_American_sea_lion/Argentina/RN−PB011/2023
EPI_ISL_18698518_A/Chicken/Argentina/1976−2/2023
EPI_ISL_18863889_A/pelican/Guatemala/23−003730−001−original/2023
EPI_ISL_19014404_A/common_grackle/Texas/24−008356−001−original/2024
EPI_ISL_19014402_A/blackbird/Texas/24−008354−001−original/2024
EPI_ISL_19014386_A/dairy_cattle/Texas/24−008749−003−original/2024
EPI_ISL_19014408_A/blackbird/Texas/24−008357−001−original/2024
EPI_ISL_19014396_A/Canada_goose/Wyoming/24−003692−001−original/2024
EPI_ISL_19014400_A/skunk/New_Mexico/24−006483−001−original/2024
EPI_ISL_19014398_A/peregrine_falcon/California/24−005915−001−original/2024
EPI_ISL_17051408_A/northern_shoveler/BC/AIVPHL−239/2022
EPI_ISL_17051398_A/canada_goose/BC/AIVPHL−226/2022
EPI_ISL_18665615_A/Great_Horned_Owl/BC/AIVPHL−1242/2023
EPI_ISL_18133390_A/goose/Vermont/USDA−012250−001/2022
EPI_ISL_18133254_A/Lesser_Scaup/Florida/USDA−009053−001/2022
EPI_ISL_18132853_A/turkey/South_Dakota/USDA−009688−001/2022
EPI_ISL_18133086_A/Backyard_bird/Michigan/USDA−010845−003/2022
EPI_ISL_16271854_A/chicken/North_Dakota/22−036126−001−original/2022
EPI_ISL_17051415_A/bald_eagle/BC/AIVPHL−263/2022
EPI_ISL_18698981_A/chicken/Missouri/23−033857−002−original/2023
EPI_ISL_17679729_A/turkey/Italy/23VIR3800−6/2023
EPI_ISL_17982390_A/Sandwich_Tern/Netherlands/4/2023
EPI_ISL_17679811_A/black−headead_gull/Spain/512−7_23VIR1687−7/2023
EPI_ISL_17606755_A/Vulpes_vulpes/Belgium/02359_0002/2023
EPI_ISL_17547373_A/European_herring_gull/Netherlands/12/2023
EPI_ISL_17710046_A/Black−Headed_Gull/England/125703/2023
Root
0.01
group
European bird
European mammal
North American bird
North American mammal
South American bird
South American mammal
STUDY

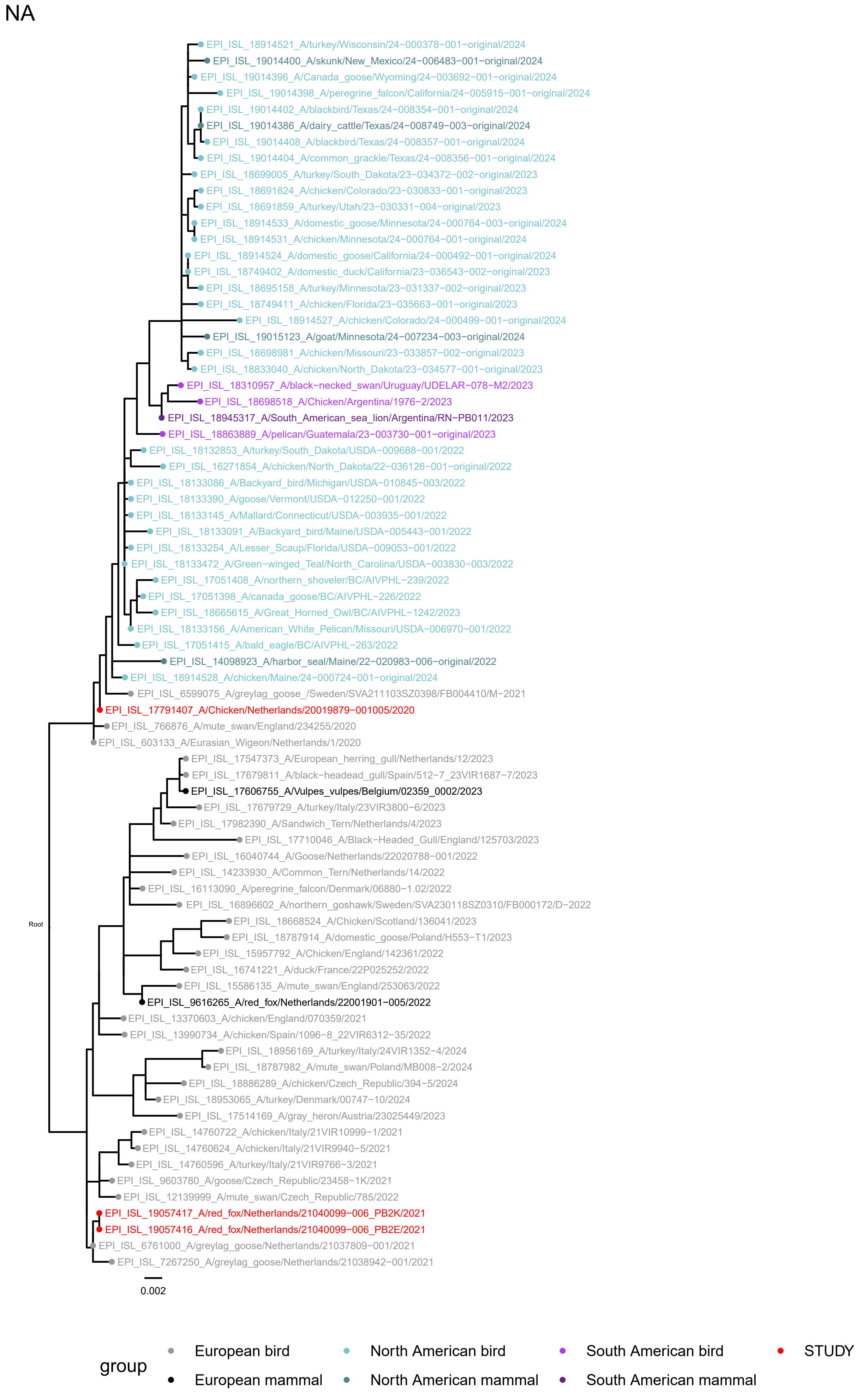
NA
EPI_ISL_18914521_A/turkey/Wisconsin/24−000378−001−original/2024
EPI_ISL_19014400_A/skunk/New_Mexico/24−006483−001−original/2024
EPI_ISL_19014396_A/Canada_goose/Wyoming/24−003692−001−original/2024
EPI_ISL_19014398_A/peregrine_falcon/California/24−005915−001−original/2024
EPI_ISL_19014402_A/blackbird/Texas/24−008354−001−original/2024
EPI_ISL_19014386_A/dairy_cattle/Texas/24−008749−003−original/2024
EPI_ISL_19014408_A/blackbird/Texas/24−008357−001−original/2024
EPI_ISL_19014404_A/common_grackle/Texas/24−008356−001−original/2024
EPI_ISL_18699005_A/turkey/South_Dakota/23−034372−002−original/2023
EPI_ISL_18691824_A/chicken/Colorado/23−030833−001−original/2023
EPI_ISL_18691859_A/turkey/Utah/23−030331−004−original/2023
EPI_ISL_18914533_A/domestic_goose/Minnesota/24−000764−003−original/2024
EPI_ISL_18914531_A/chicken/Minnesota/24−000764−001−original/2024
EPI_ISL_18914524_A/domestic_goose/California/24−000492−001−original/2024
EPI_ISL_18749402_A/domestic_duck/California/23−036543−002−original/2023
EPI_ISL_18695158_A/turkey/Minnesota/23−031337−002−original/2023
EPI_ISL_18749411_A/chicken/Florida/23−035663−001−original/2023
EPI_ISL_18914527_A/chicken/Colorado/24−000499−001−original/2024
EPI_ISL_19015123_A/goat/Minnesota/24−007234−003−original/2024
EPI_ISL_18698981_A/chicken/Missouri/23−033857−002−original/2023
EPI_ISL_18833040_A/chicken/North_Dakota/23−034577−001−original/2023
EPI_ISL_18310957_A/black−necked_swan/Uruguay/UDELAR−078−M2/2023
EPI_ISL_18698518_A/Chicken/Argentina/1976−2/2023
EPI_ISL_18945317_A/South_American_sea_lion/Argentina/RN−PB011/2023
EPI_ISL_18863889_A/pelican/Guatemala/23−003730−001−original/2023
EPI_ISL_18132853_A/turkey/South_Dakota/USDA−009688−001/2022
EPI_ISL_16271854_A/chicken/North_Dakota/22−036126−001−original/2022
EPI_ISL_18133086_A/Backyard_bird/Michigan/USDA−010845−003/2022
EPI_ISL_18133390_A/goose/Vermont/USDA−012250−001/2022
EPI_ISL_18133145_A/Mallard/Connecticut/USDA−003935−001/2022
EPI_ISL_18133091_A/Backyard_bird/Maine/USDA−005443−001/2022
EPI_ISL_18133254_A/Lesser_Scaup/Florida/USDA−009053−001/2022
EPI_ISL_18133472_A/Green−winged_Teal/North_Carolina/USDA−003830−003/2022
EPI_ISL_17051408_A/northern_shoveler/BC/AIVPHL−239/2022
EPI_ISL_17051398_A/canada_goose/BC/AIVPHL−226/2022
EPI_ISL_18665615_A/Great_Horned_Owl/BC/AIVPHL−1242/2023
EPI_ISL_18133156_A/American_White_Pelican/Missouri/USDA−006970−001/2022
EPI_ISL_17051415_A/bald_eagle/BC/AIVPHL−263/2022
EPI_ISL_14098923_A/harbor_seal/Maine/22−020983−006−original/2022
EPI_ISL_18914528_A/chicken/Maine/24−000724−001−original/2024
EPI_ISL_6599075_A/greylag_goose_/Sweden/SVA211103SZ0398/FB004410/M−2021
EPI_ISL_17791407_A/Chicken/Netherlands/20019879−001005/2020
EPI_ISL_766876_A/mute_swan/England/234255/2020
EPI_ISL_603133_A/Eurasian_Wigeon/Netherlands/1/2020
EPI_ISL_17547373_A/European_herring_gull/Netherlands/12/2023
EPI_ISL_17679811_A/black−headead_gull/Spain/512−7_23VIR1687−7/2023
EPI_ISL_17606755_A/Vulpes_vulpes/Belgium/02359_0002/2023
EPI_ISL_17679729_A/turkey/Italy/23VIR3800−6/2023
EPI_ISL_17982390_A/Sandwich_Tern/Netherlands/4/2023
EPI_ISL_17710046_A/Black−Headed_Gull/England/125703/2023
EPI_ISL_16040744_A/Goose/Netherlands/22020788−001/2022
EPI_ISL_14233930_A/Common_Tern/Netherlands/14/2022
EPI_ISL_16113090_A/peregrine_falcon/Denmark/06880−1.02/2022
EPI_ISL_16896602_A/northern_goshawk/Sweden/SVA230118SZ0310/FB000172/D−2022
Root
EPI_ISL_18668524_A/Chicken/Scotland/136041/2023
EPI_ISL_18787914_A/domestic_goose/Poland/H553−T1/2023
EPI_ISL_15957792_A/Chicken/England/142361/2022
EPI_ISL_16741221_A/duck/France/22P025252/2022
EPI_ISL_15586135_A/mute_swan/England/253063/2022
EPI_ISL_9616265_A/red_fox/Netherlands/22001901−005/2022
EPI_ISL_13370603_A/chicken/England/070359/2021
EPI_ISL_13990734_A/chicken/Spain/1096−8_22VIR6312−35/2022
EPI_ISL_18956169_A/turkey/Italy/24VIR1352−4/2024
EPI_ISL_18787982_A/mute_swan/Poland/MB008−2/2024
EPI_ISL_18886289_A/chicken/Czech_Republic/394−5/2024
EPI_ISL_18953065_A/turkey/Denmark/00747−10/2024
EPI_ISL_17514169_A/gray_heron/Austria/23025449/2023
EPI_ISL_14760722_A/chicken/Italy/21VIR10999−1/2021
EPI_ISL_14760624_A/chicken/Italy/21VIR9940−5/2021
EPI_ISL_14760596_A/turkey/Italy/21VIR9766−3/2021
EPI_ISL_9603780_A/goose/Czech_Republic/23458−1K/2021
EPI_ISL_12139999_A/mute_swan/Czech_Republic/785/2022
EPI_ISL_19057417_A/red_fox/Netherlands/21040099−006_PB2K/2021
EPI_ISL_19057416_A/red_fox/Netherlands/21040099−006_PB2E/2021
EPI_ISL_6761000_A/greylag_goose/Netherlands/21037809−001/2021
EPI_ISL_7267250_A/greylag_goose/Netherlands/21038942−001/2021
0.002
group
European bird
North American bird
South American bird
STUDY
European mammal
North American mammal
South American mammal

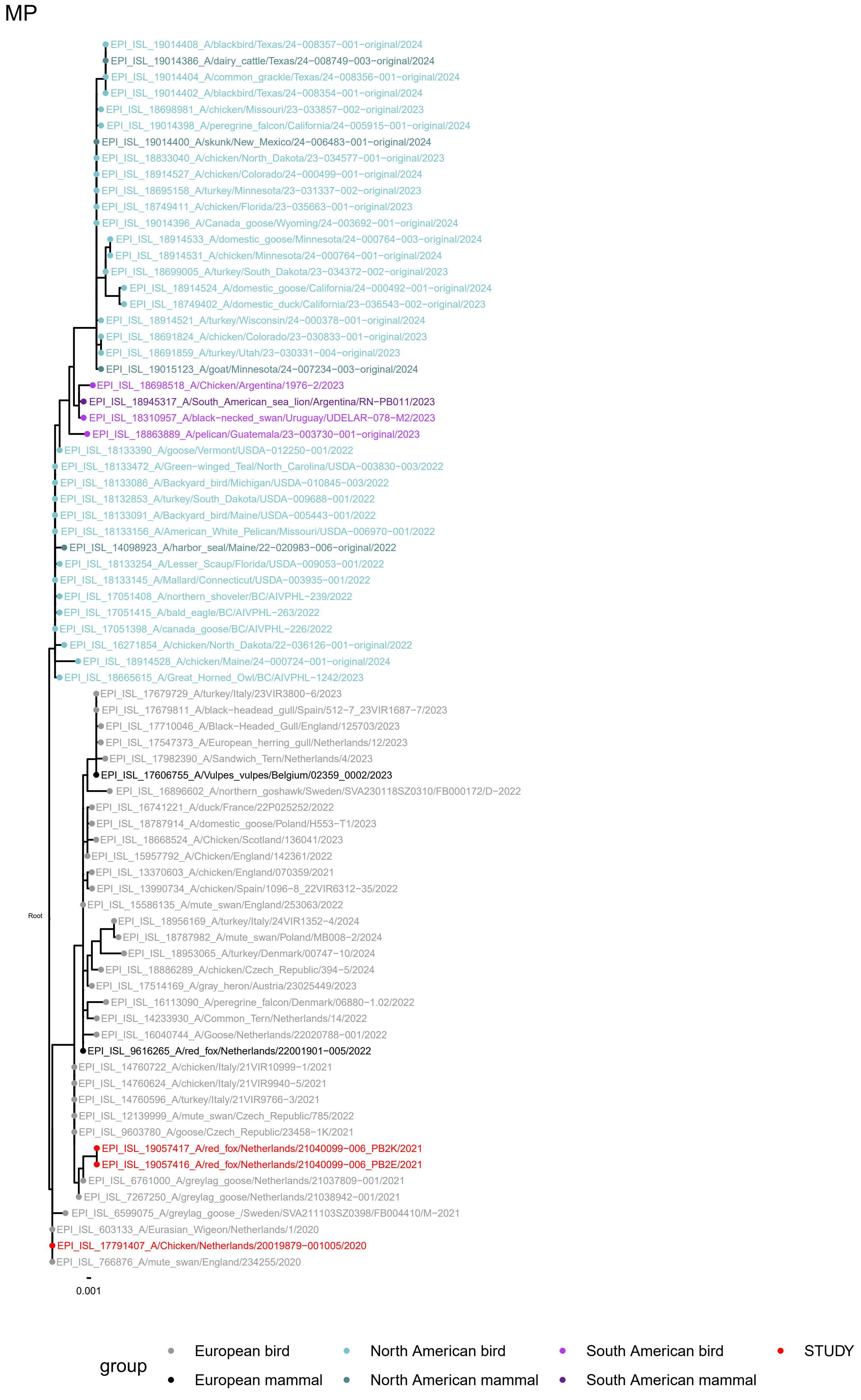
MP
EPI_ISL_19014408_A/blackbird/Texas/24−008357−001−original/2024
EPI_ISL_19014386_A/dairy_cattle/Texas/24−008749−003−original/2024
EPI_ISL_19014404_A/common_grackle/Texas/24−008356−001−original/2024
EPI_ISL_19014402_A/blackbird/Texas/24−008354−001−original/2024
EPI_ISL_18698981_A/chicken/Missouri/23−033857−002−original/2023
EPI_ISL_19014398_A/peregrine_falcon/California/24−005915−001−original/2024
EPI_ISL_19014400_A/skunk/New_Mexico/24−006483−001−original/2024
EPI_ISL_18833040_A/chicken/North_Dakota/23−034577−001−original/2023
EPI_ISL_18914527_A/chicken/Colorado/24−000499−001−original/2024
EPI_ISL_18695158_A/turkey/Minnesota/23−031337−002−original/2023
EPI_ISL_18749411_A/chicken/Florida/23−035663−001−original/2023
EPI_ISL_19014396_A/Canada_goose/Wyoming/24−003692−001−original/2024
EPI_ISL_18914533_A/domestic_goose/Minnesota/24−000764−003−original/2024
EPI_ISL_18914531_A/chicken/Minnesota/24−000764−001−original/2024
EPI_ISL_18699005_A/turkey/South_Dakota/23−034372−002−original/2023
EPI_ISL_18914524_A/domestic_goose/California/24−000492−001−original/2024
EPI_ISL_18749402_A/domestic_duck/California/23−036543−002−original/2023
EPI_ISL_18914521_A/turkey/Wisconsin/24−000378−001−original/2024
EPI_ISL_18691824_A/chicken/Colorado/23−030833−001−original/2023
EPI_ISL_18691859_A/turkey/Utah/23−030331−004−original/2023
EPI_ISL_19015123_A/goat/Minnesota/24−007234−003−original/2024
EPI_ISL_18698518_A/Chicken/Argentina/1976−2/2023
EPI_ISL_18945317_A/South_American_sea_lion/Argentina/RN−PB011/2023
EPI_ISL_18310957_A/black−necked_swan/Uruguay/UDELAR−078−M2/2023
EPI_ISL_18863889_A/pelican/Guatemala/23−003730−001−original/2023
EPI_ISL_18133390_A/goose/Vermont/USDA−012250−001/2022
EPI_ISL_18133472_A/Green−winged_Teal/North_Carolina/USDA−003830−003/2022
EPI_ISL_18133086_A/Backyard_bird/Michigan/USDA−010845−003/2022
EPI_ISL_18132853_A/turkey/South_Dakota/USDA−009688−001/2022
EPI_ISL_18133091_A/Backyard_bird/Maine/USDA−005443−001/2022
EPI_ISL_18133156_A/American_White_Pelican/Missouri/USDA−006970−001/2022
EPI_ISL_14098923_A/harbor_seal/Maine/22−020983−006−original/2022
EPI_ISL_18133254_A/Lesser_Scaup/Florida/USDA−009053−001/2022
EPI_ISL_18133145_A/Mallard/Connecticut/USDA−003935−001/2022
EPI_ISL_17051408_A/northern_shoveler/BC/AIVPHL−239/2022
EPI_ISL_17051415_A/bald_eagle/BC/AIVPHL−263/2022
EPI_ISL_17051398_A/canada_goose/BC/AIVPHL−226/2022
EPI_ISL_16271854_A/chicken/North_Dakota/22−036126−001−original/2022
EPI_ISL_18914528_A/chicken/Maine/24−000724−001−original/2024
EPI_ISL_18665615_A/Great_Horned_Owl/BC/AIVPHL−1242/2023
EPI_ISL_17679729_A/turkey/Italy/23VIR3800−6/2023
EPI_ISL_17679811_A/black−headead_gull/Spain/512−7_23VIR1687−7/2023
EPI_ISL_17710046_A/Black−Headed_Gull/England/125703/2023
EPI_ISL_17547373_A/European_herring_gull/Netherlands/12/2023
EPI_ISL_17982390_A/Sandwich_Tern/Netherlands/4/2023
EPI_ISL_17606755_A/Vulpes_vulpes/Belgium/02359_0002/2023
EPI_ISL_16896602_A/northern_goshawk/Sweden/SVA230118SZ0310/FB000172/D−2022
EPI_ISL_16741221_A/duck/France/22P025252/2022
EPI_ISL_18787914_A/domestic_goose/Poland/H553−T1/2023
EPI_ISL_18668524_A/Chicken/Scotland/136041/2023
EPI_ISL_15957792_A/Chicken/England/142361/2022
EPI_ISL_13370603_A/chicken/England/070359/2021
EPI_ISL_13990734_A/chicken/Spain/1096−8_22VIR6312−35/2022
Root
EPI_ISL_15586135_A/mute_swan/England/253063/2022
EPI_ISL_18956169_A/turkey/Italy/24VIR1352−4/2024
EPI_ISL_18787982_A/mute_swan/Poland/MB008−2/2024
EPI_ISL_18953065_A/turkey/Denmark/00747−10/2024
EPI_ISL_18886289_A/chicken/Czech_Republic/394−5/2024
EPI_ISL_17514169_A/gray_heron/Austria/23025449/2023
EPI_ISL_16113090_A/peregrine_falcon/Denmark/06880−1.02/2022
EPI_ISL_14233930_A/Common_Tern/Netherlands/14/2022
EPI_ISL_16040744_A/Goose/Netherlands/22020788−001/2022
EPI_ISL_9616265_A/red_fox/Netherlands/22001901−005/2022
EPI_ISL_14760722_A/chicken/Italy/21VIR10999−1/2021
EPI_ISL_14760624_A/chicken/Italy/21VIR9940−5/2021
EPI_ISL_14760596_A/turkey/Italy/21VIR9766−3/2021
EPI_ISL_12139999_A/mute_swan/Czech_Republic/785/2022
EPI_ISL_9603780_A/goose/Czech_Republic/23458−1K/2021
EPI_ISL_19057417_A/red_fox/Netherlands/21040099−006_PB2K/2021
EPI_ISL_19057416_A/red_fox/Netherlands/21040099−006_PB2E/2021
EPI_ISL_6761000_A/greylag_goose/Netherlands/21037809−001/2021
EPI_ISL_7267250_A/greylag_goose/Netherlands/21038942−001/2021
EPI_ISL_6599075_A/greylag_goose_/Sweden/SVA211103SZ0398/FB004410/M−2021
EPI_ISL_603133_A/Eurasian_Wigeon/Netherlands/1/2020
EPI_ISL_17791407_A/Chicken/Netherlands/20019879−001005/2020
EPI_ISL_766876_A/mute_swan/England/234255/2020
0.001
group
European bird
North American bird
South American bird
STUDY
European mammal
North American mammal
South American mammal

NS

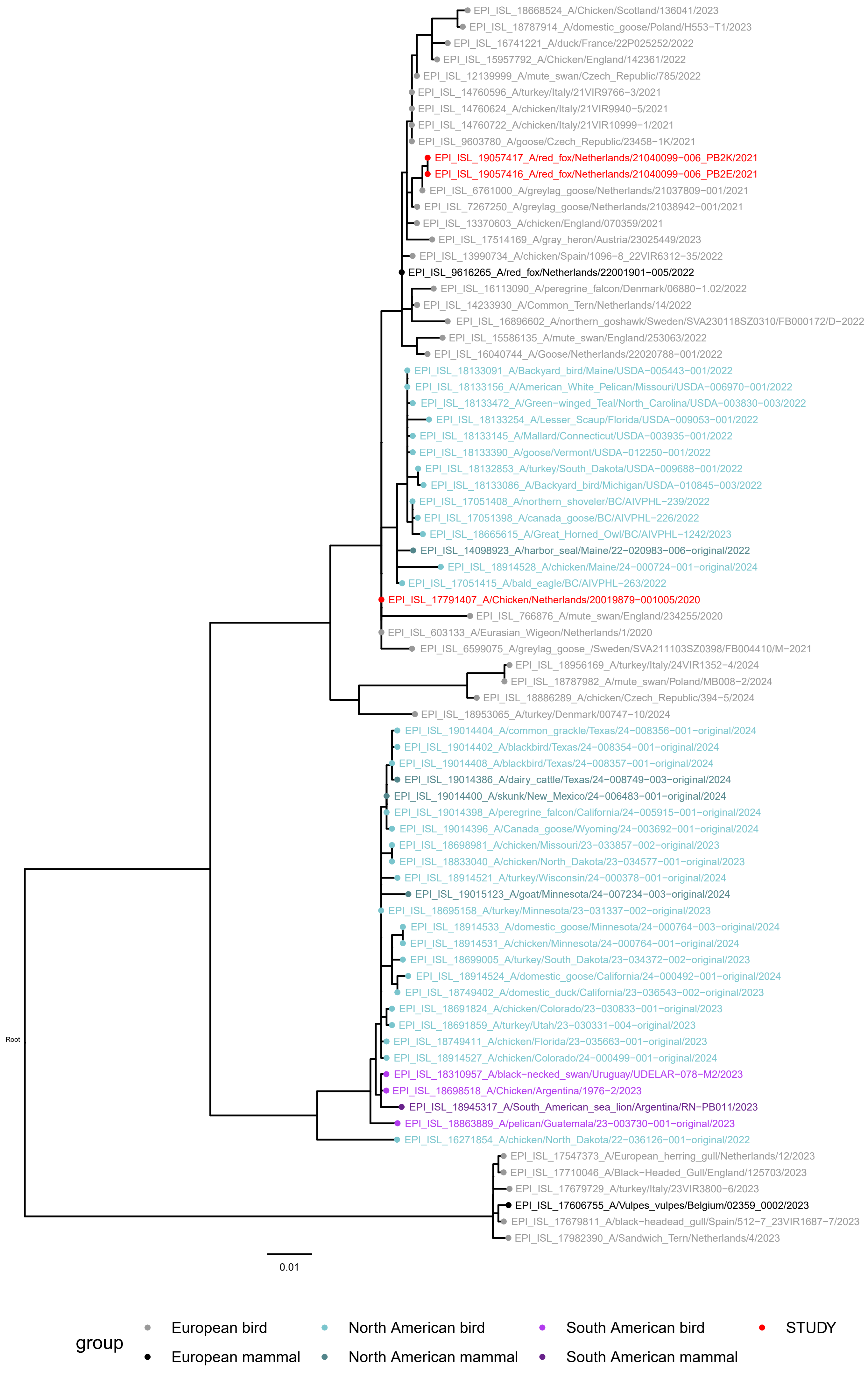
EPI_ISL_18668524_A/Chicken/Scotland/136041/2023
EPI_ISL_18787914_A/domestic_goose/Poland/H553−T1/2023
EPI_ISL_16741221_A/duck/France/22P025252/2022
EPI_ISL_15957792_A/Chicken/England/142361/2022
EPI_ISL_12139999_A/mute_swan/Czech_Republic/785/2022
EPI_ISL_14760596_A/turkey/Italy/21VIR9766−3/2021
EPI_ISL_14760624_A/chicken/Italy/21VIR9940−5/2021
EPI_ISL_14760722_A/chicken/Italy/21VIR10999−1/2021
EPI_ISL_9603780_A/goose/Czech_Republic/23458−1K/2021
EPI_ISL_19057417_A/red_fox/Netherlands/21040099−006_PB2K/2021
EPI_ISL_19057416_A/red_fox/Netherlands/21040099−006_PB2E/2021
EPI_ISL_6761000_A/greylag_goose/Netherlands/21037809−001/2021
EPI_ISL_7267250_A/greylag_goose/Netherlands/21038942−001/2021
EPI_ISL_13370603_A/chicken/England/070359/2021
EPI_ISL_17514169_A/gray_heron/Austria/23025449/2023
EPI_ISL_13990734_A/chicken/Spain/1096−8_22VIR6312−35/2022
EPI_ISL_9616265_A/red_fox/Netherlands/22001901−005/2022
EPI_ISL_16113090_A/peregrine_falcon/Denmark/06880−1.02/2022
EPI_ISL_14233930_A/Common_Tern/Netherlands/14/2022
EPI_ISL_16896602_A/northern_goshawk/Sweden/SVA230118SZ0310/FB000172/D−2022
EPI_ISL_15586135_A/mute_swan/England/253063/2022
EPI_ISL_16040744_A/Goose/Netherlands/22020788−001/2022
EPI_ISL_18133091_A/Backyard_bird/Maine/USDA−005443−001/2022
EPI_ISL_18133156_A/American_White_Pelican/Missouri/USDA−006970−001/2022
EPI_ISL_18133472_A/Green−winged_Teal/North_Carolina/USDA−003830−003/2022
EPI_ISL_18133254_A/Lesser_Scaup/Florida/USDA−009053−001/2022
EPI_ISL_18133145_A/Mallard/Connecticut/USDA−003935−001/2022
EPI_ISL_18133390_A/goose/Vermont/USDA−012250−001/2022
EPI_ISL_18132853_A/turkey/South_Dakota/USDA−009688−001/2022
EPI_ISL_18133086_A/Backyard_bird/Michigan/USDA−010845−003/2022
EPI_ISL_17051408_A/northern_shoveler/BC/AIVPHL−239/2022
EPI_ISL_17051398_A/canada_goose/BC/AIVPHL−226/2022
EPI_ISL_18665615_A/Great_Horned_Owl/BC/AIVPHL−1242/2023
EPI_ISL_14098923_A/harbor_seal/Maine/22−020983−006−original/2022
EPI_ISL_18914528_A/chicken/Maine/24−000724−001−original/2024
EPI_ISL_17051415_A/bald_eagle/BC/AIVPHL−263/2022
EPI_ISL_17791407_A/Chicken/Netherlands/20019879−001005/2020
EPI_ISL_766876_A/mute_swan/England/234255/2020
EPI_ISL_603133_A/Eurasian_Wigeon/Netherlands/1/2020
EPI_ISL_6599075_A/greylag_goose_/Sweden/SVA211103SZ0398/FB004410/M−2021
EPI_ISL_18956169_A/turkey/Italy/24VIR1352−4/2024
EPI_ISL_18787982_A/mute_swan/Poland/MB008−2/2024
EPI_ISL_18886289_A/chicken/Czech_Republic/394−5/2024
EPI_ISL_18953065_A/turkey/Denmark/00747−10/2024
EPI_ISL_19014404_A/common_grackle/Texas/24−008356−001−original/2024
EPI_ISL_19014402_A/blackbird/Texas/24−008354−001−original/2024
EPI_ISL_19014408_A/blackbird/Texas/24−008357−001−original/2024
EPI_ISL_19014386_A/dairy_cattle/Texas/24−008749−003−original/2024
EPI_ISL_19014400_A/skunk/New_Mexico/24−006483−001−original/2024
EPI_ISL_19014398_A/peregrine_falcon/California/24−005915−001−original/2024
EPI_ISL_19014396_A/Canada_goose/Wyoming/24−003692−001−original/2024
EPI_ISL_18698981_A/chicken/Missouri/23−033857−002−original/2023
EPI_ISL_18833040_A/chicken/North_Dakota/23−034577−001−original/2023
EPI_ISL_18914521_A/turkey/Wisconsin/24−000378−001−original/2024
EPI_ISL_19015123_A/goat/Minnesota/24−007234−003−original/2024
EPI_ISL_18695158_A/turkey/Minnesota/23−031337−002−original/2023
EPI_ISL_18914533_A/domestic_goose/Minnesota/24−000764−003−original/2024
EPI_ISL_18914531_A/chicken/Minnesota/24−000764−001−original/2024
EPI_ISL_18699005_A/turkey/South_Dakota/23−034372−002−original/2023
EPI_ISL_18914524_A/domestic_goose/California/24−000492−001−original/2024
EPI_ISL_18749402_A/domestic_duck/California/23−036543−002−original/2023
EPI_ISL_18691824_A/chicken/Colorado/23−030833−001−original/2023
EPI_ISL_18691859_A/turkey/Utah/23−030331−004−original/2023
EPI_ISL_18749411_A/chicken/Florida/23−035663−001−original/2023
EPI_ISL_18914527_A/chicken/Colorado/24−000499−001−original/2024
EPI_ISL_18310957_A/black−necked_swan/Uruguay/UDELAR−078−M2/2023
EPI_ISL_18698518_A/Chicken/Argentina/1976−2/2023
EPI_ISL_18945317_A/South_American_sea_lion/Argentina/RN−PB011/2023
EPI_ISL_18863889_A/pelican/Guatemala/23−003730−001−original/2023
EPI_ISL_16271854_A/chicken/North_Dakota/22−036126−001−original/2022
EPI_ISL_17547373_A/European_herring_gull/Netherlands/12/2023
EPI_ISL_17710046_A/Black−Headed_Gull/England/125703/2023
EPI_ISL_17679729_A/turkey/Italy/23VIR3800−6/2023
EPI_ISL_17606755_A/Vulpes_vulpes/Belgium/02359_0002/2023
EPI_ISL_17679811_A/black−headead_gull/Spain/512−7_23VIR1687−7/2023
EPI_ISL_17982390_A/Sandwich_Tern/Netherlands/4/2023
Root
0.01
group
European bird
European mammal
North American bird
North American mammal
South American bird
South American mammal
STUDY

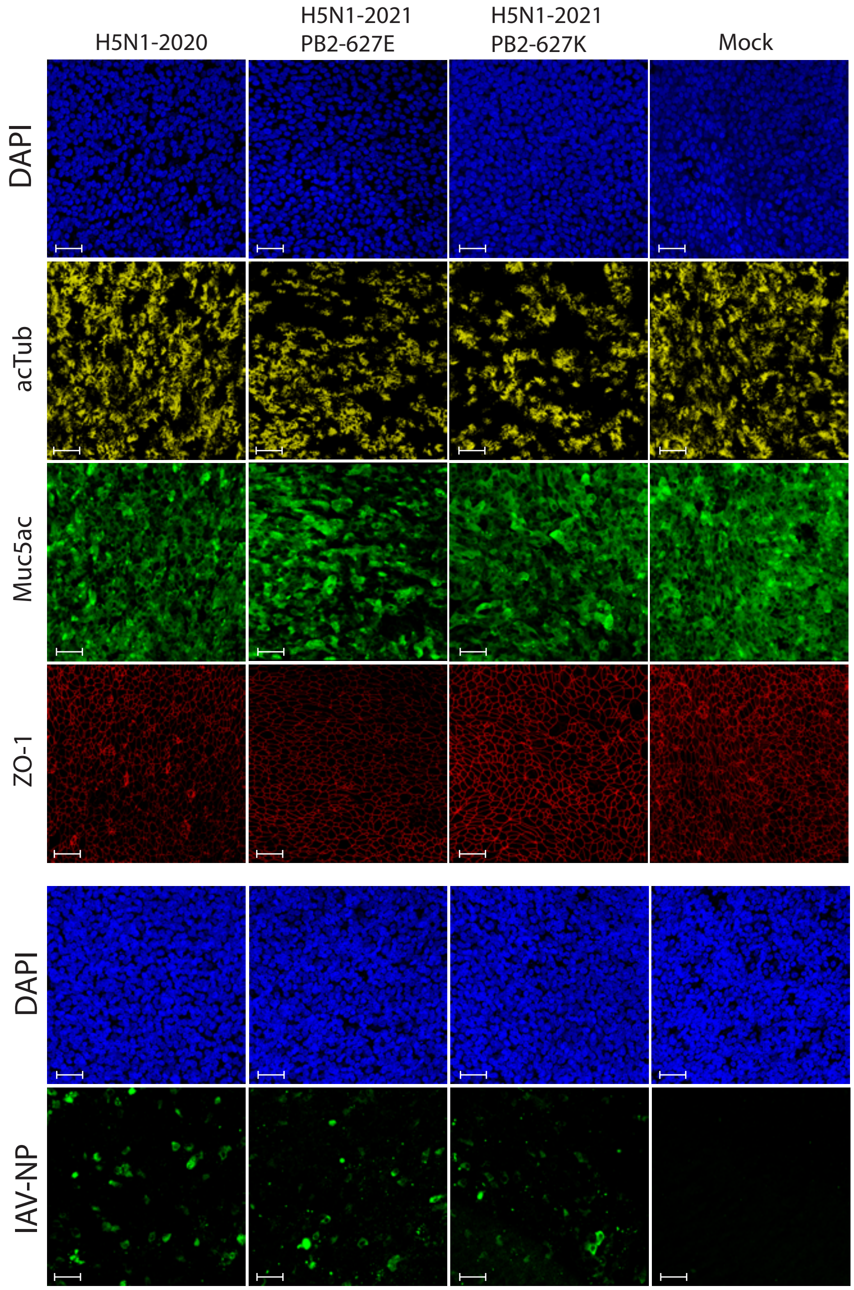

Supplementary figure 2
Four upper panels show epithelial cell markers targeting nucleic acid (DAPI, blue), acetylated tubulin (yellow), Muc5AC (green) and ZO-1 (red), indicating the presence of nuclei, ciliated cells, goblet cells and tight junctions, respectively. Two bottom panels show influenza virus (IAV) nucleoprotein (green) and subsequent counterstain with DAPI (blue), using the duplicate filters of the infection study. Images show one field of view at 40x, scalebar = 15 µm (quantification of total number of cells and influenza virus-infected cells of all fields of view are depicted in supplementary table 3).

Supplementary table 1

Table with strains obtained from the GISAID database, with acknowledgements to the original depositors. We gratefully acknowledge the authors, originating and submitting laboratories of the sequences from GISAID’s EpiFlu™ Database on which this research is based. The list is detailed below. All submitters of data may be contacted directly via www.gisaid.org

Isolate-ID	Isolate name	Country	Collection date	Originating Lab	Submitting Lab	Authors
EPI_ISL_12139999	A/mute swan/Czech Republic/785/2022	Czech Republic	2022-Jan-10	State Veterinary Institute Prague	State Veterinary Institute Prague	Alexander,Nagy;Martina,Stara;Lenka,Cernikova
EPI_ISL_13370603	A/chicken/England/070359/2021	United Kingdom	2021-Dec-13	Animal and Plant Health Agency (APHA)	Animal and Plant Health Agency (APHA)	
EPI_ISL_13990734	A/chicken/Spain/1096-8_22VIR6312-35/2022	Spain	2022-Mar-16	Laboratorio Central de Veterinaria	Istituto Zooprofilattico Sperimentale delle Venezie	Ruano, M.J.; Rocha, A.; Sanchez, A.; Aguero, M; Barbierato, G.; Zecchin, B.; Fusaro, A.; Schivo, A.; Salviato, A.; Palumbo, E.; Giussani, E.; Monne, I.; Terregino, C.
EPI_ISL_14098923	A/harbor seal/Maine/22-020983-006-original/2022	United States	2022-Jun-29	National Veterinary Services Laboratories - USDA	National Veterinary Services Laboratories - USDA	Chinh,Thanh;Franzen,Kerrie;Love, Emily;Killian,Mary;Koster,Leo;Lantz,Kristina;Torchetti,Mia;Stuber,Tod;Hicks,Jessica
EPI_ISL_14233930	A/Common Tern/Netherlands/14/2022	Netherlands	2022-Jul-14	Erasmus Medical Center	Erasmus Medical Center	
EPI_ISL_14760596	A/turkey/Italy/21VIR9766-3/2021	Italy	2021-Nov-17	Istituto Zooprofilattico Sperimentale delle Venezie, EU/OIE/Reference Laboratory and FAO Reference Centre for AI and ND	Istituto Zooprofilattico Sperimentale delle Venezie	Barbierato, G.; Zecchin, B.; Fusaro, A.; Schivo, A.; Salviato, A.; Palumbo, E.; Giussani, E.; Pastori, A.; Monne, I.; Terregino, C.
EPI_ISL_14760624	A/chicken/Italy/21VIR9940-5/2021	Italy	2021-Nov-23	Istituto Zooprofilattico Sperimentale delle Venezie, EU/OIE/Reference Laboratory and FAO Reference Centre for AI and ND	Istituto Zooprofilattico Sperimentale delle Venezie	Barbierato, G.; Zecchin, B.; Fusaro, A.; Schivo, A.; Salviato, A.; Palumbo, E.; Giussani, E.; Pastori, A.; Monne, I.; Terregino, C.
EPI_ISL_14760722	A/chicken/Italy/21VIR10999-1/2021	Italy	2021-Dec-11	Istituto Zooprofilattico Sperimentale delle Venezie, EU/OIE/Reference Laboratory and FAO Reference Centre for AI and ND	Istituto Zooprofilattico Sperimentale delle Venezie	Barbierato, G.; Zecchin, B.; Fusaro, A.; Schivo, A.; Salviato, A.; Palumbo, E.; Giussani, E.; Pastori, A.; Monne, I.; Terregino, C.
EPI_ISL_15586135	A/mute_swan/England/253063/2022	United Kingdom	2022-Sep-27	Animal and Plant Health Agency (APHA)	Animal and Plant Health Agency (APHA)	
EPI_ISL_15957792	A/Chicken/England/142361/2022	United Kingdom	2022-Oct-31	Animal and Plant Health Agency (APHA)	Animal and Plant Health Agency (APHA)	Byrne, A.; Howton, N.; Maskell, D.; Mollett, B.; Peers-Dent, J.
EPI_ISL_16040744	A/Goose/Netherlands/22020788-001/2022	Netherlands	2022-Nov-21	Wageningen Bioveterinary Research	Wageningen Bioveterinary Research	Beerens, Nancy; Harders, Frank; Pritz-Verschuren, Sylvia; Roose, Marit; Venema, Sandra; Germeraad, Evelien; Engelsma, Marc; Heutink, Rene
EPI_ISL_16113090	A/peregrine_falcon/Denmark/06880-1.02/2022	Denmark	2022-Jul-17	Statens Serumm Institute	Statens Serum Institute	Hjulsager,Charlotte; Liang,Yuan
EPI_ISL_16271854	A/chicken/North Dakota/22-036126-001-original/2022	United States	2022-Nov-08	National Veterinary Services Laboratories - USDA	National Veterinary Services Laboratories - USDA	Chinh,Thanh;Love, Emily;Franzen,Kerrie;Killian,Mary;Koster,Leo;Lantz,Kristina;Stuber, Tod;Hicks,Jessica
EPI_ISL_16741221	A/duck/France/22P025252/2022	France	2022-Dec-07	Anses (Ploufragan-Plouzané)	ANSES Agence Nationale De Securite Sanitaire De L’alimentation	
EPI_ISL_16896602	A/northern goshawk/Sweden/SVA230118SZ0310/FB000172/D-2022	Sweden	2022-Dec-28	Swedish Veterinary Agency (SVA)	Swedish Veterinary Agency (SVA)	'Siamak,Zohari'
EPI_ISL_17051398	A/canada_goose/BC/AIVPHL-226/2022	Canada	2022-Aug-15	B.C. Centre for Disease Control	British Columbia Centre for Disease Control	Himsworth, Chelsea; Prystajecky,Natalie; Tyson,John; Jassem,Agatha; Kuchinski,Kevin; Coombe, Michelle; Lee,Tracy; Azana,Rob; Fung,Janet; Chan,Michael; Cheung,Branco; Caleta,Jessica; Tsang,Frankie; Russell,Shannon; Zlosnik,James; Hoang,Linda
EPI_ISL_17051408	A/northern_shoveler/BC/AIVPHL-239/2022	Canada	2022-Sep-12	B.C. Centre for Disease Control	British Columbia Centre for Disease Control	Himsworth, Chelsea; Prystajecky,Natalie; Tyson,John; Jassem,Agatha; Kuchinski,Kevin; Coombe, Michelle; Lee,Tracy; Azana,Rob; Fung,Janet; Chan,Michael; Cheung,Branco; Caleta,Jessica; Tsang,Frankie; Russell,Shannon; Zlosnik,James; Hoang,Linda
EPI_ISL_17051415	A/bald_eagle/BC/AIVPHL-263/2022	Canada	2022-Oct-21	B.C. Centre for Disease Control	British Columbia Centre for Disease Control	Himsworth, Chelsea; Prystajecky,Natalie; Tyson,John; Jassem,Agatha; Kuchinski,Kevin; Coombe, Michelle; Lee,Tracy; Azana,Rob; Fung,Janet; Chan,Michael; Cheung,Branco; Caleta,Jessica; Tsang,Frankie; Russell,Shannon; Zlosnik,James; Hoang,Linda
EPI_ISL_17514169	A/gray_heron/Austria/23025449/2023	Austria	2023-Feb-28	Institute for Veterinary Disease Control Moedling, Austrian Agency for Health and Food Safety	Austrian Agency for Health and Food Safety (AGES)	
EPI_ISL_17547373	A/European herring gull/Netherlands/12/2023	Netherlands	2023-Apr-11	Erasmus Medical Center	Erasmus Medical Center	
EPI_ISL_17606755	A/Vulpes_vulpes/Belgium/02359_0002/2023	Belgium	2023-Mar-14	Sciensano - Animal Infectious Diseases	Sciensano, Department of Animal Infectious Diseases	Van Borm, Steven; Roupie, Virginie; Hostyn, Pierre; Mathijs, Elisabeth; Lambrecht, Benedicte; Steensels, Mieke
EPI_ISL_17679729	A/turkey/Italy/23VIR3800-6/2023	Italy	2023-Apr-24	Istituto Zooprofilattico Sperimentale delle Venezie, EU/OIE/Reference Laboratory and FAO Reference Centre for AI and ND	Istituto Zooprofilattico Sperimentale Delle Venezie	Pastori, A.; Zecchin, B.; Fusaro, A.; Schivo, A.; Salviato, A.; Palumbo, E.; Giussani, E.; Monne, I.; Terregino, C.
EPI_ISL_17679811	A/black-headead_gull/Spain/512-7_23VIR1687-7/2023	Spain	2023-Jan-31	Laboratorio Central de Veterinaria	Istituto Zooprofilattico Sperimentale Delle Venezie	Ruano,M.J.; Rocha,A.; Sanchez,A.; Aguero,M.; Pastori, A.; Zecchin, B.; Fusaro, A.; Schivo, A.; Salviato, A.; Palumbo, E.; Giussani, E.; Monne, I.; Terregino, C.
EPI_ISL_17710046	A/Black-Headed_Gull/England/125703/2023	United Kingdom	2023-May-03	Animal and Plant Health Agency (APHA)	Animal and Plant Health Agency (APHA)	
EPI_ISL_17791407	A/Chicken/Netherlands/20019879-001005/2020	Netherlands	2020-Dec-14	Wageningen Bioveterinary Research	Wageningen Bioveterinary Research	Beerens, Nancy; Harders, Frank; Pritz-Verschuren, Sylvia; Roose, Marit; Venema, Sandra; Germeraad, Evelien; Engelsma, Marc; Heutink, Rene
EPI_ISL_17982390	A/Sandwich Tern/Netherlands/4/2023	Netherlands	2023-Jun-16	Erasmus Medical Center	Erasmus Medical Center	

EPI_ISL_18132853	A/turkey/South Dakota/USDA-009688-001/2022	United States	2022-Mar-30			Youk,S., Torchetti,M.K., Lantz,K., Lenoch,J.B., Killian,M.L., Leyson,C., Bevins,S.N., Dilione,K., Ip,H.S., Stallknecht,D.E., Poulson,R.L., Suarez,D.L., Swayne,D.E.; Pantin-Jackwood,M.J.
EPI_ISL_18133086	A/Backyard bird/Michigan/USDA-010845-003/2022	United States	2022-Apr-07			Youk,S., Torchetti,M.K., Lantz,K., Lenoch,J.B., Killian,M.L., Leyson,C., Bevins,S.N., Dilione,K., Ip,H.S., Stallknecht,D.E., Poulson,R.L., Suarez,D.L., Swayne,D.E.; Pantin-Jackwood,M.J.
EPI_ISL_18133091	A/Backyard bird/Maine/USDA-005443-001/2022	United States	2022-Feb-20			Youk,S., Torchetti,M.K., Lantz,K., Lenoch,J.B., Killian,M.L., Leyson,C., Bevins,S.N., Dilione,K., Ip,H.S., Stallknecht,D.E., Poulson,R.L., Suarez,D.L., Swayne,D.E.; Pantin-Jackwood,M.J.
EPI_ISL_18133145	A/Mallard/Connecticut/USDA-003935-001/2022	United States	2022-Feb-03			Youk,S., Torchetti,M.K., Lantz,K., Lenoch,J.B., Killian,M.L., Leyson,C., Bevins,S.N., Dilione,K., Ip,H.S., Stallknecht,D.E., Poulson,R.L., Suarez,D.L., Swayne,D.E.; Pantin-Jackwood,M.J.
EPI_ISL_18133156	A/American White Pelican/Missouri/USDA-006970-001/2022	United States	2022-Feb-28			Youk,S., Torchetti,M.K., Lantz,K., Lenoch,J.B., Killian,M.L., Leyson,C., Bevins,S.N., Dilione,K., Ip,H.S., Stallknecht,D.E., Poulson,R.L., Suarez,D.L., Swayne,D.E.; Pantin-Jackwood,M.J.
EPI_ISL_18133254	A/Lesser Scaup/Florida/USDA-009053-001/2022	United States	2022-Mar-18			Youk,S., Torchetti,M.K., Lantz,K., Lenoch,J.B., Killian,M.L., Leyson,C., Bevins,S.N., Dilione,K., Ip,H.S., Stallknecht,D.E., Poulson,R.L., Suarez,D.L., Swayne,D.E.; Pantin-Jackwood,M.J.
EPI_ISL_18133390	A/goose/Vermont/USDA-012250-001/2022	United States	2022-Apr-18			Youk,S., Torchetti,M.K., Lantz,K., Lenoch,J.B., Killian,M.L., Leyson,C., Bevins,S.N., Dilione,K., Ip,H.S., Stallknecht,D.E., Poulson,R.L., Suarez,D.L., Swayne,D.E.; Pantin-Jackwood,M.J.
EPI_ISL_18133472	A/Green-winged Teal/North Carolina/USDA-003830-003/2022	United States	2022-Jan-29			Youk,S., Torchetti,M.K., Lantz,K., Lenoch,J.B., Killian,M.L., Leyson,C., Bevins,S.N., Dilione,K., Ip,H.S., Stallknecht,D.E., Poulson,R.L., Suarez,D.L., Swayne,D.E.; Pantin-Jackwood,M.J.
EPI_ISL_18310957	A/black-necked swan/Uruguay/UDELAR-078-M2/2023	Uruguay	2023-Mar-15			Marandino,A., Tomas,G., Panzera,Y., Leizagoyen,C., Perez,R., Bassetti,L., Negro,R., Rodriguez,S.; Perez,R.
EPI_ISL_18665615	A/Great_Horned_Owl/BC/AIVPHL-1242/2023	Canada	2023-Jul-24	B.C. Centre for Disease Control	Public Health Agency of Canada	Himsworth, Chelsea; Prystajecky,Natalie; Tyson,John; Jassem,Agatha; Kuchinski,Kevin; Coombe, Michelle; Lee,Tracy; Azana,Rob; Fung,Janet; Chan,Michael; Cheung,Branco; Caleta,Jessica; Tsang,Frankie; Yang,Kevin; Russell,Shannon; Zlosnik,James; Hoang,Linda
EPI_ISL_18668524	A/Chicken/Scotland/136041/2023	United Kingdom	2023-Nov-21	Animal and Plant Health Agency (APHA)	Animal and Plant Health Agency (APHA)	
EPI_ISL_18691824	A/chicken/Colorado/23-030833-001-original/2023	United States	2023-Oct-11	National Veterinary Services Laboratories - USDA	National Veterinary Services Laboratories - USDA	Franzen,Kerrie;Love,Emily;Ozella,Mikaela;Killian,Mary;Lantz,Kristina;Stuber,Tod;Hicks,Jessica;Norris,Cameron
EPI_ISL_18691859	A/turkey/Utah/23-030331-004-original/2023	United States	2023-Oct-05	National Veterinary Services Laboratories - USDA	National Veterinary Services Laboratories - USDA	Franzen,Kerrie;Love,Emily;Ozella,Mikaela;Killian,Mary;Lantz,Kristina;Stuber,Tod;Hicks,Jessica;Norris,Cameron
EPI_ISL_18695158	A/turkey/Minnesota/23-031337-002-original/2023	United States	2023-Oct-15	National Veterinary Services Laboratories - USDA	National Veterinary Services Laboratories - USDA	Franzen,Kerrie;Love,Emily;Ozella,Mikaela;Killian,Mary;Lantz,Kristina;Stuber,Tod;Hicks,Jessica;Norris,Cameron
EPI_ISL_18698518	A/Chicken/Argentina/1976-2/2023	Argentina	2023-May-01	Dirección del Laboratorio Animal, Dirección General de Laboratorios y Control Técnico, SENASA.	Instituto Nacional de Enfermedades Infecciosas Dr. C.G. Malbrán	Artuso, M.; Marchione, V.; Benedetti, E.; Chamorro, A.; Bonastre, P.; Alvarez, A.; Piccini, L.; Ponde, A.; Barrios, E.; Fabeiro, M.; Waisman, K.; Coppola, L.; Popklepovich, T.; Pontoriero, A.; Riva, D.
EPI_ISL_18698981	A/chicken/Missouri/23-033857-002-original/2023	United States	2023-Nov-02	National Veterinary Services Laboratories - USDA	National Veterinary Services Laboratories - USDA	Franzen,Kerrie;Love,Emily;Ozella,Mikaela;Killian,Mary;Lantz,Kristina;Stuber,Tod;Hicks,Jessica;Norris,Cameron
EPI_ISL_18699005	A/turkey/South Dakota/23-034372-002-original/2023	United States	2023-Nov-07	National Veterinary Services Laboratories - USDA	National Veterinary Services Laboratories - USDA	Franzen,Kerrie;Love,Emily;Ozella,Mikaela;Killian,Mary;Lantz,Kristina;Stuber,Tod;Hicks,Jessica;Norris,Cameron
EPI_ISL_18749402	A/domestic duck/California/23-036543-002-original/2023	United States	2023-Nov-15	National Veterinary Services Laboratories - USDA	National Veterinary Services Laboratories - USDA	Franzen,Kerrie;Love,Emily;Ozella,Mikaela;Killian,Mary;Lantz,Kristina;Stuber,Tod;Hicks,Jessica;Norris,Cameron
EPI_ISL_18749411	A/chicken/Florida/23-035663-001-original/2023	United States	2023-Nov-15	National Veterinary Services Laboratories - USDA	National Veterinary Services Laboratories - USDA	Franzen,Kerrie;Love,Emily;Ozella,Mikaela;Killian,Mary;Lantz,Kristina;Stuber,Tod;Hicks,Jessica;Norris,Cameron
EPI_ISL_18787914	A/domestic_goose/Poland/H553-T1/2023	Poland	2023-Dec-20	National Veterinary Research Institut Poland, PIWet-PIB	National Veterinary Research Institut Poland, PIWet-PIB	Swieton E., Smietanka K.
EPI_ISL_18787982	A/mute_swan/Poland/MB008-2/2024	Poland	2024-Jan-03	National Veterinary Research Institut Poland, PIWet-PIB	National Veterinary Research Institut Poland, PIWet-PIB	Swieton E., Smietanka K.
EPI_ISL_18833040	A/chicken/North Dakota/23-034577-001-original/2023	United States	2023-Nov-08	National Veterinary Services Laboratories - USDA	National Veterinary Services Laboratories - USDA	Franzen,Kerrie;Love,Emily;Ozella,Mikaela;Killian,Mary;Lantz,Kristina;Stuber,Tod;Hicks,Jessica;Norris,Cameron
EPI_ISL_18863889	A/pelican/Guatemala/23-003730-001-original/2023	Guatemala	2023-Jan-27	National Veterinary Services Laboratories - USDA	National Veterinary Services Laboratories - USDA	Cordon y Cordon,Julio;Serrano,Lucero;Rodas,Jorge;Franzen,Kerrie;Love,Emily;Ozella,Mikaela;Killian,Mary;Lantz,Kristina;Stuber,Tod;Hicks,Jessica;Norris,Cameron
EPI_ISL_18886289	A/chicken/Czech_Republic/394-5/2024	Czech Republic	2024-Jan-04	State Veterinary Institute Prague	State Veterinary Institute Prague	Nagy,Alexander;Cernikova,Lenka;Klickova,Eliska;Haban,Lubomir;Sterbova,Michaela;Orenicova,Kristina
EPI_ISL_18914521	A/turkey/Wisconsin/24-000378-001-original/2024	United States	2024-Jan-03	National Veterinary Services Laboratories - USDA	National Veterinary Services Laboratories - USDA	Franzen,Kerrie;Love,Emily;Ozella,Mikaela;Killian,Mary;Lantz,Kristina;Stuber,Tod;Hicks,Jessica;Norris,Cameron
EPI_ISL_18914524	A/domestic goose/California/24-000492-001-original/2024	United States	2024-Jan-03	National Veterinary Services Laboratories - USDA	National Veterinary Services Laboratories - USDA	Franzen,Kerrie;Love,Emily;Ozella,Mikaela;Killian,Mary;Lantz,Kristina;Stuber,Tod;Hicks,Jessica;Norris,Cameron
EPI_ISL_18914527	A/chicken/Colorado/24-000499-001-original/2024	United States	2024-Jan-04	National Veterinary Services Laboratories - USDA	National Veterinary Services Laboratories - USDA	Franzen,Kerrie;Love,Emily;Ozella,Mikaela;Killian,Mary;Lantz,Kristina;Stuber,Tod;Hicks,Jessica;Norris,Cameron

EPI_ISL_18914528	A/chicken/Maine/24-000724-001-original/2024	United States	2024-Jan-08	National Veterinary Services Laboratories - USDA	National Veterinary Services Laboratories - USDA	Franzen,Kerrie;Love,Emily;Ozella,Mikaela;Killian,Mary;Lantz,Kristina;Stuber,Tod;Hicks,Jessica;Norris,Cameron
EPI_ISL_18914531	A/chicken/Minnesota/24-000764-001-original/2024	United States	2024-Jan-04	National Veterinary Services Laboratories - USDA	National Veterinary Services Laboratories - USDA	Franzen,Kerrie;Love,Emily;Ozella,Mikaela;Killian,Mary;Lantz,Kristina;Stuber,Tod;Hicks,Jessica;Norris,Cameron
EPI_ISL_18914533	A/domestic goose/Minnesota/24-000764-003-original/2024	United States	2024-Jan-04	National Veterinary Services Laboratories - USDA	National Veterinary Services Laboratories - USDA	Franzen,Kerrie;Love,Emily;Ozella,Mikaela;Killian,Mary;Lantz,Kristina;Stuber,Tod;Hicks,Jessica;Norris,Cameron
EPI_ISL_18945317	A/South American sea lion/Argentina/RN-PB011/2023	Argentina	2023-Aug-26	Instituto Nacional de Tecnologia Agropecuaria, Instituto de Virologia e Innovaciones Tecnologicas		Rimondi,A., Vanstreels,R.E.T., Olivera,V.S., Donini,A., Miqueo Lauriente,M.; Uhart,M.
EPI_ISL_18953065	A/turkey/Denmark/00747-10/2024	Denmark	2024-Jan-27	Statens Serum Institut	Statens Serum Institute	Hjulsager,C; Liang, Y
EPI_ISL_18956169	A/turkey/Italy/24VIR1352-4/2024	Italy	2024-Feb-20	Istituto Zooprofilattico Sperimentale delle Venezie, EU/OIE/Reference Laboratory and FAO Reference Centre for AI and ND	Istituto Zooprofilattico Sperimentale Delle Venezie	Cavicchio, L.; Zecchin, B.; Fusaro, A.; Schivo, A.; Salviato, A.; Palumbo, E.; Giussani, E.; Pastori, A.; Monne, I.; Terregino, C.
EPI_ISL_19014386	A/dairy cattle/Texas/24-008749-003-original/2024	United States	2024-Mar-20	National Veterinary Services Laboratories - USDA	National Veterinary Services Laboratories - USDA	Aufderhar,Matthew;Franzen,Kerrie;Love,Emily;Killian,Mary;Lantz,Kristina;Stuber,Tod;Hicks,Jessica;Norris,Cameron
EPI_ISL_19014396	A/Canada goose/Wyoming/24-003692-001-original/2024	United States	2024-Jan-25	National Veterinary Services Laboratories - USDA	National Veterinary Services Laboratories - USDA	Aufderhar,Matthew;Franzen,Kerrie;Love,Emily;Killian,Mary;Lantz,Kristina;Stuber,Tod;Hicks,Jessica;Norris,Cameron
EPI_ISL_19014398	A/peregrine falcon/California/24-005915-001-original/2024	United States	2024-Feb-14	National Veterinary Services Laboratories - USDA	National Veterinary Services Laboratories - USDA	Aufderhar,Matthew;Franzen,Kerrie;Love,Emily;Killian,Mary;Lantz,Kristina;Stuber,Tod;Hicks,Jessica;Norris,Cameron
EPI_ISL_19014400	A/skunk/New Mexico/24-006483-001-original/2024	United States	2024-Feb-23	National Veterinary Services Laboratories - USDA	National Veterinary Services Laboratories - USDA	Aufderhar,Matthew;Franzen,Kerrie;Love,Emily;Killian,Mary;Lantz,Kristina;Stuber,Tod;Hicks,Jessica;Norris,Cameron
EPI_ISL_19014402	A/blackbird/Texas/24-008354-001-original/2024	United States	2024-Mar-16	National Veterinary Services Laboratories - USDA	National Veterinary Services Laboratories - USDA	Aufderhar,Matthew;Franzen,Kerrie;Love,Emily;Killian,Mary;Lantz,Kristina;Stuber,Tod;Hicks,Jessica;Norris,Cameron
EPI_ISL_19014404	A/common grackle/Texas/24-008356-001-original/2024	United States	2024-Mar-18	National Veterinary Services Laboratories - USDA	National Veterinary Services Laboratories - USDA	Aufderhar,Matthew;Franzen,Kerrie;Love,Emily;Killian,Mary;Lantz,Kristina;Stuber,Tod;Hicks,Jessica;Norris,Cameron
EPI_ISL_19014408	A/blackbird/Texas/24-008357-001-original/2024	United States	2024-Mar-16	National Veterinary Services Laboratories - USDA	National Veterinary Services Laboratories - USDA	Aufderhar,Matthew;Franzen,Kerrie;Love,Emily;Killian,Mary;Lantz,Kristina;Stuber,Tod;Hicks,Jessica;Norris,Cameron
EPI_ISL_19015123	A/goat/Minnesota/24-007234-003-original/2024	United States	2024-Mar-08	National Veterinary Services Laboratories - USDA	National Veterinary Services Laboratories - USDA	Aufderhar,Matthew;Franzen,Kerrie;Love,Emily;Killian,Mary;Lantz,Kristina;Stuber,Tod;Hicks,Jessica;Norris,Cameron
EPI_ISL_19057416	A/red fox/Netherlands/21040099-006_PB2E/2021	Netherlands	2021-Dec-03	Wageningen Bioveterinary Research	Wageningen Bioveterinary Research	Beerens, Nancy; Harders, Frank; Pritz-Verschuren, Sylvia; Roose, Marit; Venema, Sandra; Germeraad, Evelien; Engelsma, Marc; Heutink, Rene
EPI_ISL_19057417	A/red fox/Netherlands/21040099-006_PB2K/2021	Netherlands	2021-Dec-03	Wageningen Bioveterinary Research	Wageningen Bioveterinary Research	Beerens, Nancy; Harders, Frank; Pritz-Verschuren, Sylvia; Roose, Marit; Venema, Sandra; Germeraad, Evelien; Engelsma, Marc; Heutink, Rene
EPI_ISL_603133	A/Eurasian Wigeon/Netherlands/1/2020	Netherlands	2020-Oct-16	Erasmus Medical Center	Erasmus Medical Center	
EPI_ISL_6599075	A/greylag goose /Sweden/SVA211103SZ0398/FB004410/M-2021	Sweden	2021-Nov-01	Swedish Veterinary Agency (SVA)	Swedish Veterinary Agency (SVA)	
EPI_ISL_6761000	A/greylag goose/Netherlands/21037809-001/2021	Netherlands	2021-Oct-31	Wageningen Bioveterinary Research	Wageningen Bioveterinary Research	Beerens, Nancy; Harders, Frank; Pritz-Verschuren, Sylvia; Roose, Marit; Germeraad, Evelien; Engelsma, Marc; Heutink, Rene
EPI_ISL_7267250	A/greylag goose/Netherlands/21038942-001/2021	Netherlands	2021-Nov-15	Wageningen Bioveterinary Research	Wageningen Bioveterinary Research	Beerens, Nancy; Harders, Frank; Pritz-Verschuren, Sylvia; Roose, Marit; Venema, Sandra; Germeraad, Evelien; Engelsma, Marc; Heutink, Rene
EPI_ISL_766876	A/mute_swan/England/234255/2020	United Kingdom	2020-Dec-03	Animal and Plant Health Agency (APHA)	Animal and Plant Health Agency (APHA)	
EPI_ISL_9603780	A/goose/Czech Republic/23458-1K/2021	Czech Republic	2021-Nov-26	State Veterinary Institute Prague	State Veterinary Institute Prague	Alexander,Nagy;Lenka,Cernikova;Martina,Stara
EPI_ISL_9616265	A/red fox/Netherlands/22001901-005/2022	Netherlands	2022-Jan-27	Wageningen Bioveterinary Research	Wageningen Bioveterinary Research	Beerens, Nancy; Harders, Frank; Pritz-Verschuren, Sylvia; Roose, Marit; Venema, Sandra; Germeraad, Evelien; Engelsma, Marc; Heutink, Rene
EPI_ISL_94331	A/duck/Eastern China/108/2008	China	2008-Dec-15			Gu,M.; Liu,W.; Zhao,G.; Xu,Q.; Zhang,W.; Zhang,X.; Cao,Y.; Li,Y.; P
EPI_ISL_163493	A/Sichuan/26221/2014	China	2014-Apr-21		WHO Chinese National Influenza Center	
EPI_ISL_171695	A/Baikal teal/Korea/H52/2014	Korea, Republic of	2014-Jan-20			Jeong,J.; Kang,H.M.; Lee,E.K.; Song,B.M.; Kwon,Y.K.; Kim,H.R.; Ch
EPI_ISL_173878	A/gyrfalcon/Washington/41088-6/2014	United States	2014-Dec-08			Ip,H.S.; Kim-Torchetti,M.; Crespo,R.; Kohrs,P.; DeBruyn,P.; Mansfie
EPI_ISL_256213	A/Hubei/29578/2016	China	2016-Apr-15		WHO Chinese National Influenza Center	

Supplementary table 2

Mean infectious virus titers determined by EID50 and TCID50. The dosage for each WD-AEC was determined using the EID50 and the TCID50 dosage was determined retrospectively. The sum of the total amount of collected infectious virus particles on 4hpi, 24hpi, 48hpi and 72hpi is calculated for each filter separately.

	**H5N1-2020**	**H5N1-2021 PB2E**	**H5N1-2021 PB2K**
**Stock log10 EID_{50}/ml**	9.85	9.10	9.34
**Stock log10 $TCID_{50}$/ml**	8.67	8.67	8.17
**Log10 EID_{50} inoculum titer**	6.00	6.00	6.00
**Log10 $TCID_{50}$ inoculum titer**	4.82	5.61	4.85
**Total amount of infectious virus particles (log 10 TCID50/0.5ml, volume of each wash)**	4.77	6.57	6.27

Supplementary table 3
Average number of ciliated cells and goblet cells are determined for eight representative fields of view at 40x magnification by making a maximum projection of the entire z-stack, subtracting the background and thresholding the images. The background subtracted, thresholded ZO-1 staining is used as a mask to identify cell borders. Total number of nuclei and virus positive cells are counted on background subtracted, thresholded images (without ZO-1 mask). Cells are automatically counted using ImageJ version 2. 14.0 (macro can be made available upon request)

		H5N1-2020	H5N1-2021 PB2-627E	H5N1-2021 PB2-627K	Mock
**specific staining**	Mean [SD] DAPI	842.3 [85.6]	838.6 [55.4]	791 [73.2]	654.1 [94.6]
	Mean [SD] Muc5Ac	122.1 [37.5]	154.4 [49.4]	148.3 [40.1]	158.5 [26.2]
	Mean [SD] acTub	347.5 [73.5]	264.4 [130.7]	302.5 [150.0]	269.9 [142.5]
**IAV-NP staining**	Mean [SD] DAPI	731.6 [62.1]	706.0 [53.0]	916.0 [175.6]	693.0 [103.7]
	Mean [SD] IAV-NP	24.0 [8.2]	6.6 [3.7]	7.6 [4.2]	0 [0.0]
